# The ETHYLENE RESPONSE FACTOR6-*GRETCHEN HAGEN3.5* module regulates rooting and heat tolerance in *Dimocarpus longan*

**DOI:** 10.1093/plphys/kiaf096

**Published:** 2025-03-19

**Authors:** Xueying Zhang, Shuting Zhang, Shuangjie Wang, Wentao Ma, Tingkai Zhai, Jie Gao, Chunwang Lai, Zihao Zhang, Yukun Chen, Zhongxiong Lai, Yuling Lin

**Affiliations:** Institute of Horticultural Biotechnology, Fujian Agriculture and Forestry University, Fuzhou, Fujian 350002, China; Key Laboratory of Genetics, Breeding and Multiple Utilization of Crops, Ministry of Education, Fujian Agriculture and Forestry University, Fuzhou, Fujian 350002, China; Institute of Horticultural Biotechnology, Fujian Agriculture and Forestry University, Fuzhou, Fujian 350002, China; Key Laboratory of Genetics, Breeding and Multiple Utilization of Crops, Ministry of Education, Fujian Agriculture and Forestry University, Fuzhou, Fujian 350002, China; Institute of Horticultural Biotechnology, Fujian Agriculture and Forestry University, Fuzhou, Fujian 350002, China; Key Laboratory of Genetics, Breeding and Multiple Utilization of Crops, Ministry of Education, Fujian Agriculture and Forestry University, Fuzhou, Fujian 350002, China; Institute of Horticultural Biotechnology, Fujian Agriculture and Forestry University, Fuzhou, Fujian 350002, China; Key Laboratory of Genetics, Breeding and Multiple Utilization of Crops, Ministry of Education, Fujian Agriculture and Forestry University, Fuzhou, Fujian 350002, China; Institute of Horticultural Biotechnology, Fujian Agriculture and Forestry University, Fuzhou, Fujian 350002, China; Key Laboratory of Genetics, Breeding and Multiple Utilization of Crops, Ministry of Education, Fujian Agriculture and Forestry University, Fuzhou, Fujian 350002, China; Institute of Horticultural Biotechnology, Fujian Agriculture and Forestry University, Fuzhou, Fujian 350002, China; Key Laboratory of Genetics, Breeding and Multiple Utilization of Crops, Ministry of Education, Fujian Agriculture and Forestry University, Fuzhou, Fujian 350002, China; Institute of Horticultural Biotechnology, Fujian Agriculture and Forestry University, Fuzhou, Fujian 350002, China; Key Laboratory of Genetics, Breeding and Multiple Utilization of Crops, Ministry of Education, Fujian Agriculture and Forestry University, Fuzhou, Fujian 350002, China; Institute of Horticultural Biotechnology, Fujian Agriculture and Forestry University, Fuzhou, Fujian 350002, China; Key Laboratory of Genetics, Breeding and Multiple Utilization of Crops, Ministry of Education, Fujian Agriculture and Forestry University, Fuzhou, Fujian 350002, China; Institute of Horticultural Biotechnology, Fujian Agriculture and Forestry University, Fuzhou, Fujian 350002, China; Key Laboratory of Genetics, Breeding and Multiple Utilization of Crops, Ministry of Education, Fujian Agriculture and Forestry University, Fuzhou, Fujian 350002, China; Institute of Horticultural Biotechnology, Fujian Agriculture and Forestry University, Fuzhou, Fujian 350002, China; Key Laboratory of Genetics, Breeding and Multiple Utilization of Crops, Ministry of Education, Fujian Agriculture and Forestry University, Fuzhou, Fujian 350002, China; Institute of Horticultural Biotechnology, Fujian Agriculture and Forestry University, Fuzhou, Fujian 350002, China; Key Laboratory of Genetics, Breeding and Multiple Utilization of Crops, Ministry of Education, Fujian Agriculture and Forestry University, Fuzhou, Fujian 350002, China

## Abstract

Heat stress can seriously affect plant growth and development. Ethylene response factors (ERFs) play important roles in plant development and physiological responses. Here, we identified DlERF6, an ERF family transcription factor that promotes heat tolerance in *Dimocarpus longan*. *DlERF6* was strongly induced by heat stress and IAA treatment in longan roots. Overexpression of *DlERF6* generated abundant, fast-growing hairy roots and enhanced longan heat stress tolerance by promoting IAA biosynthesis and reactive oxygen species (ROS) scavenging. Additional assays indicated that DlERF6 directly binds to the *DlGH3.5* promoter and represses its expression. Overexpressing *DlGH3.5* reduced hairy root number, root length, and heat tolerance, concomitant with a reduction in IAA content and ROS scavenging. Collectively, these results reveal the molecular mechanism through which the DlERF6–*DlGH3.5* module regulates root growth and heat stress tolerance, providing a gene network that can be used for the genetic improvement of longan.

## Teaser

An ethylene response factor regulates longan root growth and heat tolerance by modulating reactive oxygen species and auxin homeostasis.

## Introduction

The increasing global temperature has adversely affected the development of plants worldwide, especially that of subtropical fruiting plants (Fahad et al. 2017). Plants have evolved a series of defense mechanisms to reduce damage induced by thermal stress, accompanied by changes in morphological, physiological, and biochemical characteristics (Ma et al. 2021; Ren et al. 2021; Chen et al. 2022; Guihur et al. 2022). For example, well-developed root systems can enhance the tolerance of plants to abiotic stress (Wang et al. 2024). Morphological changes in root architecture impact the nutrient and water uptake efficiency of plants (Van Norman et al. 2013; Seo et al. 2020). In rice (*Oryza* sativa), wheat (*Triticum aestivum*), and *Brassica oleracea*, a well-developed root system and fast root expansion improve plant heat tolerance (Kilasi et al. 2018; Boter et al. 2023).

Ethylene response factors (ERFs) regulate plant growth and responses to abiotic stresses (Xie et al. 2019; Feng et al. 2020). For example, *AtERF1* inhibits primary root elongation in Arabidopsis by regulating auxin and ethylene accumulation (Mao et al. 2016). *AtERF109* promotes root formation by regulating auxin signaling (Cai et al. 2014), and *AtERF109* is induced by wounding and promotes auxin biosynthesis (Ye et al. 2020). Additionally, it was found that MdERF114 directly regulates *Peroxidase 63* (*PRX63*) to promote the accumulation of lignin and increase *Fusarium solani* resistance by using *Agrobacterium rhizogenes*-mediated root transformation (Liu et al. 2023). In *Oryza sativa*, the ERF subfamily gene *OsDREB1A* improves drought, salt, and cold tolerance (Dubouzet et al. 2003). In Arabidopsis, *DREB2A* enhances plant heat stress tolerance (Mizoi et al. 2012). Moreover, *DREB2C* improves heat stress tolerance by activating the transcription of heat shock-related genes (Singh and Laxmi 2015; Mizoi et al. 2019). These findings demonstrate that ERF transcription factors (TFs) play crucial roles in plant growth and stress response. However, there are no reports on the involvement of ERF proteins in root growth and heat tolerance in *Dimocarpus longan*.

Auxin biosynthesis and transport play a role in root formation and plant responses to abiotic stress (Marhavý et al. 2013). As reported, heat stress promotes auxin biosynthesis and transport in roots to reduce heat injury (Hanzawa et al. 2013). Auxin biosynthesis mutants *taa1* and *yuc8* reduce hypocotyl growth in response to heat stress (Sun et al. 2012). Moreover, GRETCHEN HAGEN3 (GH3) family genes encode indole 3-acetic acid (IAA)-amido synthetases, which conjugate IAA to amino acids (Mellor et al. 2016). GH3 family genes play important roles in plant growth and stress response (Singh et al. 2015). In Arabidopsis, *GH3.3*, *GH3.5,* and *GH3.6* regulate the growth of adventitious roots (Sorin et al. 2006; Gutierrez et al. 2012). In apple trees, *MsGH3.5* negatively regulates root growth by reducing IAA accumulation (Yuan et al. 2013; Zhao et al. 2020). Moreover, *MsGH3.6*-overexpressing apple trees generated *via A*. *rhizogenes*-mediated transformation of hairy roots presented a reduced IAA content, inhibited root growth, and decreased resistance to drought stress (Geng et al. 2019; Jiang et al. 2022). In Arabidopsis, GH3 genes inhibit auxin synthesis in response to cold stress (Yuan et al. 2013). In rice, *GH3-2* decreases IAA levels and reduces drought resistance (Du et al. 2012). However, in cotton, *GH3.5* positively regulates drought and salt tolerance (Kirungu et al. 2019), indicating that GH3 genes regulate plant growth and stress responses *via* distinct mechanisms in different plants. In pear trees, PuERF2 increases *PuGH3.1* expression to regulate IAA metabolism during fruit ripening (Yue et al. 2019). AcERF1B and AcERF073 induce *AcGH3.1* expression to promote the degradation of free IAA, which in turn accelerates postharvest kiwifruit ripening (Gan et al. 2021). However, the regulatory mechanism in longan involving ERF TFs and GH3 genes warrants further investigation.

The overproduction of reactive oxygen species (ROS) can cause lipid peroxidation, resulting in the degradation of cell membranes, proteins, and nucleic acids and affecting plant survival (Zhang et al. 2016). Accordingly, plants produce enzymatic and nonenzymatic antioxidants to eliminate ROS. Superoxide dismutase (SOD), catalase (CAT), peroxidase (POD), and glutathione transferase (GST) are the major antioxidant enzymes involved in the plant heat response (Almeselmani et al. 2009). Nonenzymatic antioxidants, such as flavonoids and phenylpropanoids, can increase ROS scavenging under heat stress conditions (Escandón et al. 2018). In addition, the environmental stress response involves crosstalk between ROS and plant hormones (Tiwari et al. 2022). A high level of ROS inhibits the expression of auxin-related genes, thereby inhibiting plant growth and development (Jia et al. 2016). Moreover, an increase in extracellular ROS levels reduces the expression of auxin receptor Aux/IAA transcriptional repressors (Blomster et al. 2011).

Longan (*Dimocarpus longan*) is a widely cultivated and economically important fruit crop in tropical and subtropical regions. However, heat stress can damage the leaves, flower organs, and fruit tissue of longan, causing an imbalance in cell homeostasis and inhibiting growth and development. Therefore, a better understanding of the heat resistance mechanisms of longan may contribute to the development of cultivars with high heat stress tolerance. ERF TFs play important roles in plant development and stress response. However, the functions of ERFs in longan remain unclear, especially in root growth and the response to heat stress. Here, the potential participation of DlERF6 in the response of longan to heat stress was investigated. In this study, we characterized an ERF TF, DlERF6, which positively regulates longan root growth and resistance to heat stress. Under heat stress treatment, *DlERF6* positively regulated ROS detoxification, auxin homeostasis, and flavonoid and lignin biosynthesis in longan. In addition, *DlERF6* regulated the ubiquitination of proteins involved in primary and secondary metabolism and antioxidation under heat stress, thereby affecting longan heat resistance. In contrast, the silencing of *DlERF6* repressed root growth and decreased heat tolerance in longan. We further revealed that *DlGH3.5* is a direct target gene of DlERF6 and a negative regulator of root growth and heat stress tolerance in longan. These results demonstrate the crucial role of the DlERF6–*DlGH3.5* regulatory network in plant root growth and heat tolerance, providing insights for the artificial breeding of longan varieties with heat resistance.

## Results

### DlERF6 is localized in the nucleus and responsive to heat and IAA treatment

ERF TFs play important roles in plant development and responses to abiotic stresses. In a previous study, we identified a longan ERF TF, DlERF6, which is involved in the heat response of longan somatic embryos (Zhang et al. 2023). The RNA-seq results revealed that *DlERF6* was expressed across all tissues; moreover, it was dominantly expressed in stems and roots, followed by seeds and young fruits (Supplementary Fig. S1A). We also performed the expression profile of *DlERF6* after heat and IAA treatment *via* RT-qPCR. Following heat stress (42 °C), the expression of *DlERF6* increased about 11.13-fold (at 24 h) and 17.71-fold (at 48 h). In addition, the expression of *DlERF6* was increased about 2.61-fold (at 24 h) and 7.94-fold (at 48 h) under IAA (1 mg·L^−1^) treatment (Supplementary Fig. S1, B and C, Supplementary Table S1), indicating that *DlERF6* was upregulated by heat stress and IAA treatment in longan roots. Promoter analysis revealed that the *DlERF6* promoter contains 1 auxin response element, 2 high-temperature response elements, and 1 drought response element (Supplementary Fig. S1F).

DlERF6 contains a conserved AP2 domain and a 999-bp open reading frame (ORF) and encodes a protein of 332 amino acids. Phylogenetic analysis revealed that DlERF6 is homologous to AtERF5 and AtERF6 (Supplementary Fig. S1, D and E). Subcellular localization analysis suggested that DlERF6 was localized in the nucleus (Supplementary Fig. S1G).

### DlERF6 positively regulates longan hairy root growth

**Figure 1.**
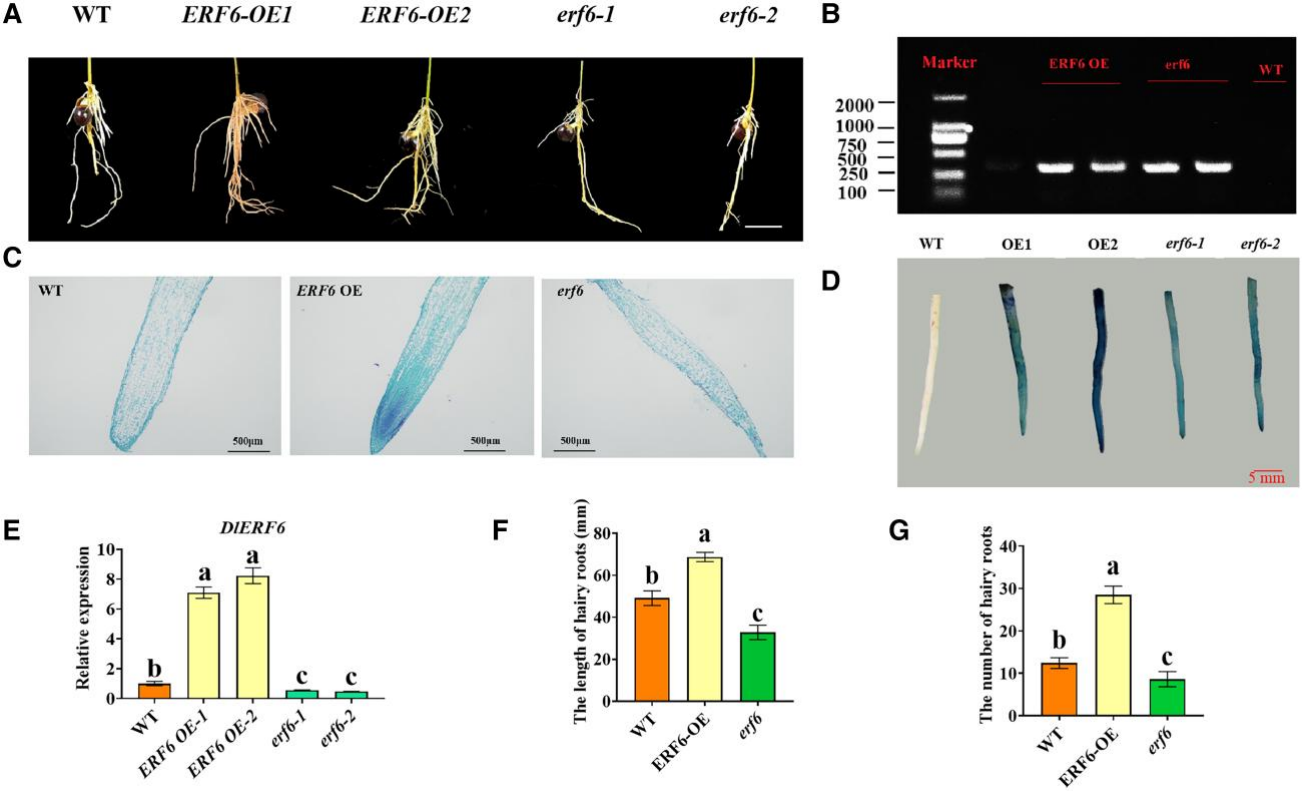
Phenotypic and molecular characterization of *DlERF6* transgenic roots. **A)** The phenotype of *DlERF6* transgenic roots. Bars = 5 cm. Images were digitally extracted for comparison. **B)** PCR analysis for GUS in independent transgenic hairy roots. **C)** Longitudinal section of root tip region of hairy roots of wild-type (WT) and *DlERF6* transgenic lines. Roots were collected, in paraffin section, and observed with 0.5% toluidine blue staining. **D)** GUS staining of the wild-type (WT) and *DlERF6* transgenic roots. Images were digitally extracted for comparison. **E)** RT-qPCR analysis of *DlERF6* in wild-type (WT) and transgenic roots. Overexpression (OE) 1, 2, different *DlERF6*-overexpressing roots. RNAi (*erf1*, *erf2*), different *DlERF6*-RNAi roots. One-way ANOVA was performed. Error bars represent the Sd of mean values (*n* = 3), and significant differences (*P* < 0.05) between groups are indicated by “a”, “b” and “c” . Images were digitally extracted for comparison. **F)** The statistical results of the hairy roots length of Wild-Type (WT) and *DlERF6* OE, RNAi transgenic lines. Error bars represent the Sd of mean values (*n* = 3), and significant differences (*P* < 0.05) between groups are indicated by “a,” “b,” and “c.” **G)** The statistical results of the hairy roots numbers of wild-type (WT) and *DlERF*6 OE, RNAi transgenic lines. One-way ANOVA was performed. Error bars represent the Sd of mean values (*n* = 10), and significant differences (*P* < 0.05) between groups are indicated by “a,” “b,” and “c.”

To elucidate the function of *DlERF6* in longan, *DlERF6* was overexpressed in longan *via* a transgenic approach or silenced *via* RNA interference (RNAi) (Fig. 1A). Because the pCAMBIA-1301 and pTCK303 vectors carry the β-glucuronidase (GUS) reporter gene, GUS staining and PCR were performed to confirm that the construct was successfully inserted into the longan genome (Fig. 1, B and D). The RT-qPCR results further revealed that the expression level of *DlERF6* in the roots of *DlERF6*-OE lines was greater than that in wild-type (WT) plants and that the expression level of *DlERF6* was lower in *DlERF6*-RNAi (*erf1* and *erf2*) lines (Fig. 1E, Supplementary Table S2). As shown in Fig. 1, F and G, the *DlERF6*-OE lines presented longer and denser roots than the WT plants, and the number and length of the *DlERF6*-OE lines roots were almost 130% and 42% greater than those of the WT plant roots, respectively. In contrast, the number and length of the *DlERF6-*RNAi lines roots decreased relative to those of the WT plants (Supplementary Tables S3 and S4). Compared with those of the WT plants' root tips, the meristematic areas of the *DlERF6*-OE lines root tips were larger, and the cells were arrayed more neatly and closely. However, the cells in the *DlERF6-*RNAi line root tips were not closely and neatly arranged (Fig. 1C). These results showed that *DlERF6* plays a positive role in the regulation of longan roots architecture, length, and number.

### *DlERF6-*overexpressing plants exhibit increased tolerance to heat stress

**Figure 2.**
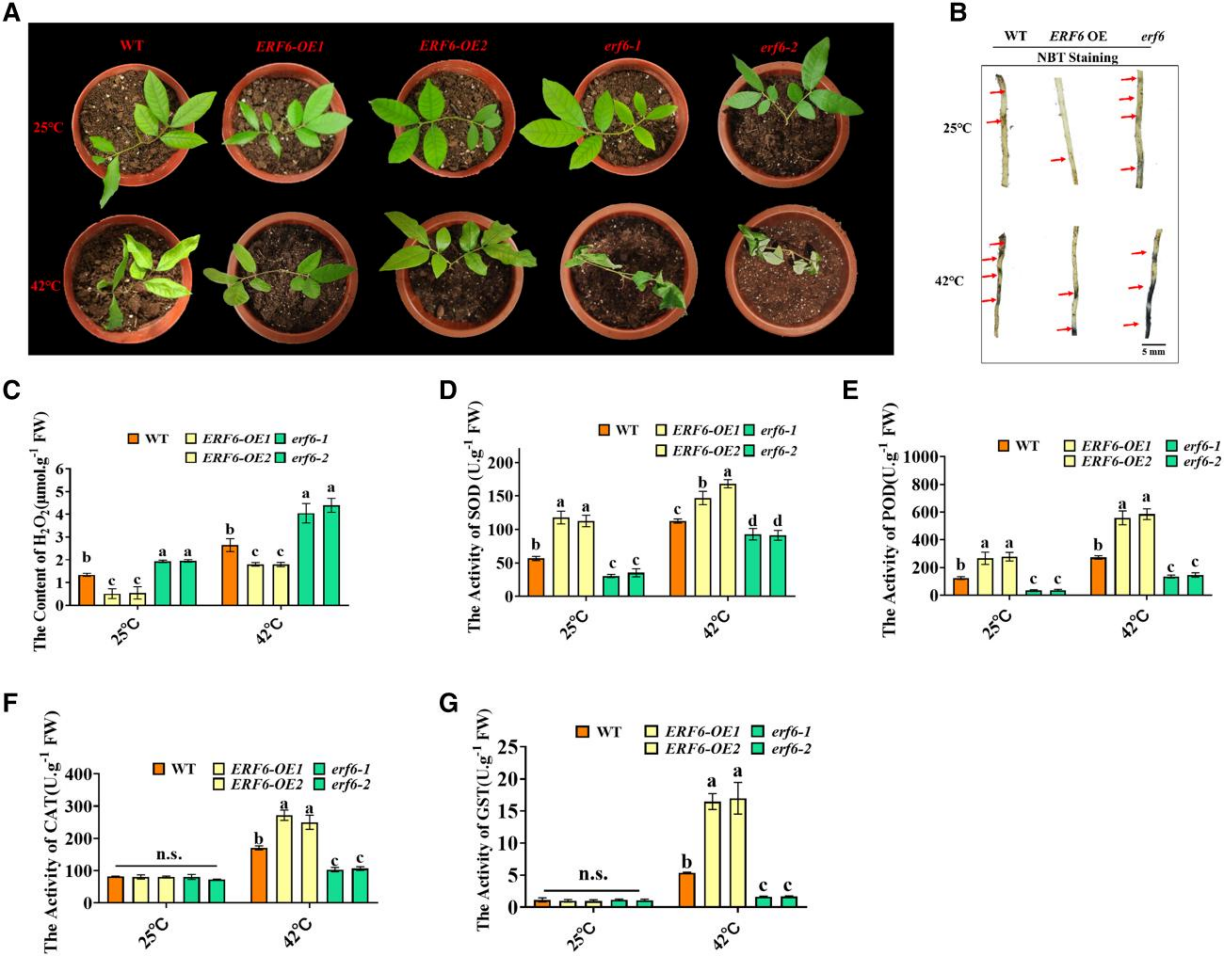
*DlERF6* overexpression plants are more tolerant to heat stress. **A)** Phenotype of plants wild-type (WT), *DlERF6* overexpressing (OE), and RNAi in the roots under heat treatment. Scale bar = 5 cm. Images were digitally extracted for comparison. **B)** Nitroblue tetrazolium (NBT) was used to detect the accumulation of superoxide and hydrogen peroxide (H_2_O_2_) in wild-type (WT) and *DlERF6* transgenic roots. The arrow indicates the staining site. Images were digitally extracted for comparison. Images were digitally extracted for comparison. **C)** The H_2_O_2_ content of wild-type (WT), *DlERF6* transgenic roots under normal and heat stress treatment. FW: Fresh weight. Error bars represent the Sd of mean values (*n* = 3), and significant differences (*P* < 0.05) between groups are indicated by “a,” “b,” and “c.” **D–G)** The SOD, POD, CAT, and GST activities of wild-type (WT) and *DlERF6* transgenic roots under normal and heat treatment. FW: Fresh weight. One-way ANOVA was performed. Error bars represent the Sd of mean values (*n* = 3), and significant differences (*P* < 0.05) between groups are indicated by “a,” “b,” and “c.”

Our initial analysis revealed that *DlERF6* is induced by heat treatment in longan (Supplementary Fig. S1B). To identify the function of *DlERF6*, 2 OE transgenic lines (*DlERF6*-OE1 and *DlERF6*-OE2) with high expression levels of *DlERF6* and 2 RNAi lines (*erf1* and *erf2*) were obtained and selected for further analysis. We exposed the transgenic and WT lines to heat stress (42 °C) for 3d. All the plants grew well under non-stress conditions and presented similar phenotypes. Under heat stress, the *DlERF6-OE* lines presented the greatest tolerance to heat conditions, presented only minimal heat damage, maintained a healthy color, and showed no apparent signs of wilting (Fig. 2A). However, the *DlERF6*-RNAi lines developed severe thermosensitive symptoms, and leaves showed curling and yellowing wilting, indicating that functional loss of *DlERF6* inhibited the growth of longan under heat stress. Moreover, nitroblue tetrazolium (NBT) staining was performed to assess the accumulation of superoxide and H_2_O_2_ in the *DlERF6-*OE, WT, and *DlERF6-*RNAi lines roots, revealing obvious accumulation of superoxide and H_2_O_2_ in the *DlERF6-*RNAi lines roots compared with the WT roots under normal and heat stress conditions (Fig. 2B).

In addition to the marked phenotypic differences, the H_2_O_2_ content was evidently lower in the *DlERF6*-OE lines roots and leaves than in the WT plants under heat stress conditions (Fig. 2C, Supplementary Fig. S2A). The activities of SOD, POD, CAT, and GST were greater in the *DlERF6*-OE roots and leaves than in the WT roots and leaves (Fig. 2, D to G, Supplementary Fig. S2, B to E, Supplementary Table S5) under heat stress condition. In contrast, the *DlERF6*-RNAi lines presented a lower ability to scavenge ROS, with the *erf1* and *erf2* lines showing higher H_2_O_2_ contents than the WT plants but lower antioxidant enzyme activities than the WT plants, resulting in more severe oxidative stress damage under heat stress condition (Fig. 2, D to G, Supplementary Fig. S2, B to E, Supplementary Table S5). These results suggest that *DlERF6* positively regulates heat stress tolerance in longan by enhancing the function of the antioxidant system.

### Transcriptional regulation of the heat stress response in *DlERF6*-OE lines

The heat response is regulated by a range of response genes. After 3d of heat stress, 4,513 genes were upregulated and 5,587 genes were downregulated in the *DlERF6*-OE roots compared with the WT roots. Gene Ontology (GO) enrichment analysis revealed that RNA modification, the extracellular region, the defense response, the intracellular membrane-bound organelle and the ethylene-activated signaling pathway were enriched in the differentially expressed genes (DEGs) (Supplementary Fig. S3A). Kyoto Encyclopedia of Genes and Genomes (KEGG) enrichment analysis revealed that starch and sucrose metabolism, plant hormone signal transduction, phenylpropanoid biosynthesis, carotenoid biosynthesis, flavonoid biosynthesis, and lsoflavonoid biosynthesis were enriched in the DEGs (Supplementary Fig. S3B). These results suggest the involvement of membrane-bound organelles, biological processes, plant hormone signal transduction, and primary and secondary metabolism in the heat stress response of *DlERF6*-OE lines.

Heat stress produces ROS, which cause severe oxidative damage to plants (Slimen et al. 2014). In this study, *DlERF6* increased ROS scavenging ability in longan roots under heat stress. We further identified ROS signaling-related DEGs in *DlERF6*-OE lines under high-temperature conditions (Supplementary Table S6), among which 56 genes were upregulated and 19 genes were downregulated (Supplementary Fig. S3, C and D). The upregulated oxidant enzyme-encoding genes included 4 SOD genes, 24 POD genes, 21 GST genes, 3 glutathione reductase (GSH) genes, and 4 ascorbate peroxidase (APX) genes (Banerjee et al. 2010). These increased expression levels of genes encoding antioxidant enzymes in the *DlERF6*-OE roots may promote the scavenging of excessive amounts of ROS induced by heat stress.

Autophagy helps plants remove toxic protein aggregates and damaged organelles under stress (Ding et al. 2008). In this study, 13 autophagy-related DEGs were identified (Supplementary Fig. S3E, Supplementary Table S6), 11 genes were upregulated, and 2 genes were downregulated in the *DlERF6*-OE lines under heat stress compared with the WT lines. Under heat stress, heat shock proteins (HSPs) are also activated to alleviate or prevent heat damage and help scavenge accumulated ROS (Zhu et al. 2020a; Guo et al. 2022). In this study, 4 differentially expressed HSP genes were identified, among which 3 genes were upregulated in the *DlERF6*-OE lines under heat stress compared with the WT plants (Supplementary Fig. S3E, Supplementary Table S6). The upregulation of these genes may increase the heat tolerance of *DlERF6*-OE lines by protecting proteins from denaturation and aggregation (Pang et al. 2020).

TFs play crucial roles in the regulatory network of heat stress responses in plants. After heat treatment, we analyzed the expression patterns of NAM/ATAF/CUC domain transcription factor, basic helix-loop helix, and ERF TFs in the *DlERF6*-OE and WT lines (Supplementary Fig. S3, F to H, Supplementary Table S7), and a series of TFs were identified. These TFs may be involved in the response to heat stress in *DlERF6*-OE lines.

### DlERF6 regulates auxin metabolism in longan

**Figure 3.**
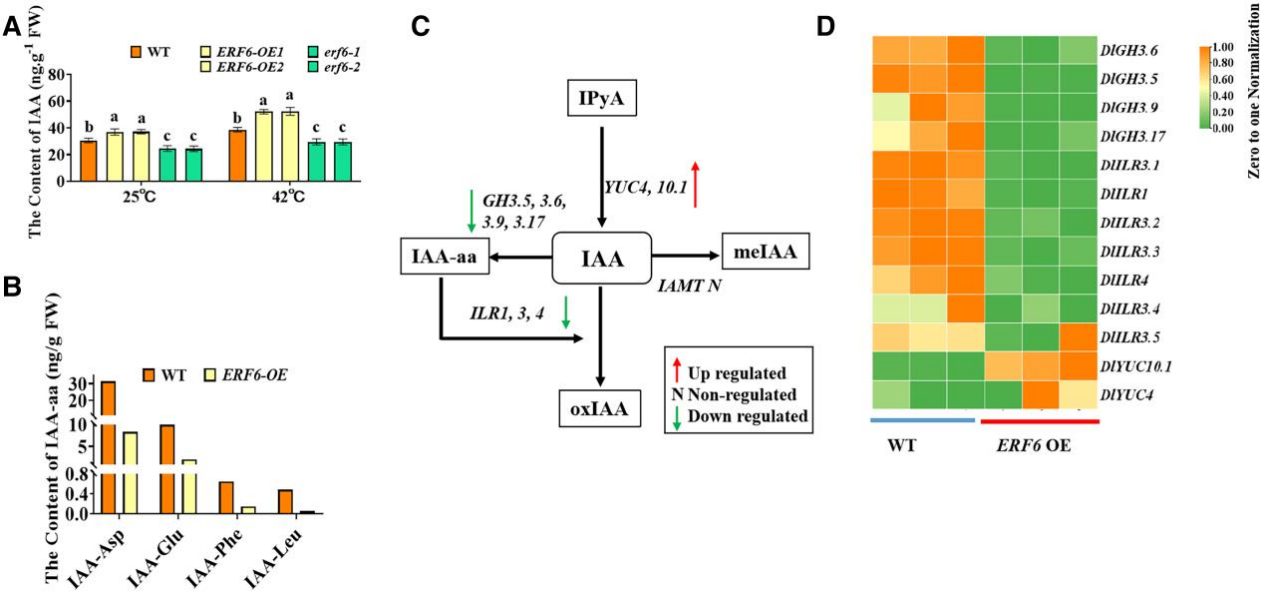
Expression of auxin metabolism genes in *DlERF6* overexpression roots in response to heat stress. **A)** The IAA (Indole-3-Acetic Acid) content of wild-type (WT), *DlERF6* transgenic roots under normal and heat stress. FW: Fresh weight. One-way ANOVA was performed. Error bars represent the Sd of mean values (*n* = 3), and significant differences (*P* < 0.05) between groups are indicated by “a,” “b,” and “c.” **B)** Quantification of IAA-amino acid (IAA-aa) conjugates in wild-type (WT), *DlERF6* overexpression roots under heat stress (*n* = 1). FW: Fresh weight. **C)** Diagram of the auxin biosynthesis and conjugate pathways in *DlERF6* overexpression (OE) roots in response to heat stress. The downward arrows, upward arrows, and N(ns) indicate the downregulated genes, upregulated genes, and non-regulated genes respectively in *DlERF6* overexpression (OE) roots. The black frame represents different auxin components. **D)** Heatmap showing the relative expression level of genes involved in auxin metabolism in *DlERF6* overexpression (OE) roots in response to heat stress.

The auxin signaling pathway plays essential roles in the regulation of root growth and the abiotic stress response in plants (Parveen and Rahman 2021; Ai et al. 2023; Song et al. 2024), and ERF family genes are involved in auxin biosynthesis (Ye et al. 2020). In this study, the IAA content of the *DlERF6*-RNAi roots was lower than that of the WT roots under both normal and heat stress conditions, although heat stress increased IAA levels in both the WT and *DlERF6*-RNAi lines (Fig. 3A). The higher IAA content of the *DlERF6*-OE lines may have contributed to their heat stress resistance. Furthermore, the levels of IAA-Asp, IAA-Glu, IAA-Phe, and IAA-Leu decreased in the *DlERF6*-OE roots under heat stress (Fig. 3B).

Among the DEGs, auxin signaling-related genes showed changes in expression in *DlERF6*-OE roots under heat stress (Fig. 3, C and D). The upregulation of genes involved in auxin biosynthesis and the downregulation of genes involved in auxin conjugation and degradation might be involved in the mediation of auxin metabolism by *DlERF6* under heat stress (Fig. 3C). In summary, the above results indicate that the IAA content is positively regulated by *DlERF6*, thus amplifying auxin signaing and promoting heat defense in longan.

### DlERF6 positively regulates longan fitness in response to heat stress by promoting lignin and flavonoid metabolite biosynthesis

Among the phenylpropanoid metabolic pathways, flavonoid and lignin metabolic pathways are usually activated by abiotic stress to protect against oxidative stress (Hichri et al. 2011; Xu et al. 2015; Choi et al. 2023). ERFs are involved in the regulation of flavonoid and lignin biosynthesis in plant abiotic stress responses (Zhang et al. 2020; Wu et al. 2022). In this study, flavonoid biosynthesis and lignin biosynthetic processes were among the top 20 enriched pathways in the DEGs in the *DlERF6*-OE lines subjected to heat stress conditions (Supplementary Fig. S3, A and B). Among the DEGs, several flavonoid and lignin biosynthesis-related key genes were identified (Supplementary Fig. S4A), including *DlPAL*, *Dl4CL*, *DlF3′H*, *DlF3′5'H*, *DlF3H*, *DlCHS*, *DlUFGT*, *DlLAR*, *DlCCR*, *DlCAD*, *DlCSE*, *DlCOMT*, and *DlCCoAOMT* (Vanholme et al. 2019; Liu et al. 2021). We indicated that *DlERF6* might regulate flavonoid and lignin biosynthesis and positively regulate longan fitness in response to heat stress.

We further asked whether the levels of metabolites were altered in *DlERF6*-OE lines under heat stress. A total of 6,330 metabolites were upregulated and 1,824 metabolites were downregulated in the *DlERF6*-OE roots compared with the WT roots under heat stress. KEGG analysis revealed that amino acid metabolism, phenylalanine metabolism, phenylpropanoid biosynthesis, and flavonoid biosynthesis were enriched in the metabolites whose abundance increased (Supplementary Fig. S4B, Supplementary Tables S8 and S9). Amino acid metabolism, galactose metabolism, and starch and sucrose metabolism were enriched in the metabolites whose expression decreased (Supplementary Fig. S4C). These results further confirmed the important role of phenylalanine metabolism in the heat stress response of *DlERF6*-OE lines. Compared with those in the WT plants, the abundances of 36 flavonoid metabolites in the *DlERF6*-OE lines were increased, and the abundances of 2 flavonoid metabolites were decreased (Supplementary Fig. S4D). Twelve lignin metabolites were increased and 3 were decreased in the *DlERF6*-OE lines compared with the WT (Supplementary Fig. S4D). The increased lignin and flavonoid metabolites in *DlERF6*-OE lines may improve ROS scavenging under heat stress. These results suggested the positive regulation of lignin and flavonoid metabolites by *DlERF6* under heat stress.

### DNA affinity purification sequencing (DAP-seq) analysis of DlERF6 target genes

To identify downstream target genes of DlERF6, we further constructed gDNA libraries with *DlERF6*-OE roots as the materials and performed DAP-seq. A total of 29,592 peaks were identified in the genome via 2 technical replicates (Supplementary Fig. S5A). The reads were distributed mainly on Chr1 and Chr11 (Supplementary Fig. S5B). A total of 4,022 (13.59%) peaks were located within the 2 kb upstream promoter regions. In addition, 796 (2.69%), 379 (1.28%), 6,593 (22.28%), 7,857 (26.55%), and 1,429 (4.83%) genes were located within the 5′UTR, 3′ UTR, exons, introns, and downstream, respectively (Supplementary Fig. S5C). A heatmap revealed a general trend of greater binding intensity within the vicinity of the transcription start site among the genes, indicating the influence of DlERF6 on transcription initiation (Supplementary Fig. S5D). We further analyzed the binding motifs of the DlERF6 protein, one DNA motif, GCC-box (“GCCGCC”), with strong significance was identified (E value = 1.1e−004) (Supplementary Fig. S5E). The binding site motif has also been identified in apples and Populus (Kong et al. 2023; Liu et al. 2023).

TFs mainly regulate gene transcription by directly binding to target gene promoters, thus, genes with promoter-binding sites were selected for further analysis. GO enrichment analysis revealed that developmental processes, such as growth, the regulation of biological processes, hormone metabolism and response, and defense responses, such as antioxidant activity, immune system processes, and responses to stimuli, were enriched in the genes whose promoters bind DlERF6 (Supplementary Fig. S5F). The KEGG pathway enrichment analysis revealed that hormone signal transduction, ascorbate and aldarate metabolism, ubiquitin-mediated proteolysis, glycolysis/gluconeogenesis, and amino acid metabolism were enriched in the promoter-binding genes (Supplementary Fig. S5G). In summary, DlERF6 may play important roles in metabolic processes, development and defense responses in longan.

### Overexpression of *DlERF6* alters protein ubiquitination in longan under heat stress

Ubiquitination is involved in DNA repair and protein quality control to regulate plant cell survival and cell death (Kerscher et al. 2006). Under heat stress, the ubiquitin–proteasome system is activated to clear misfolded and unfolded proteins and restore cellular metabolism (Chen and Yin 2011; Hwang and Qi 2018). According to the DAP-Seq data, the KEGG analysis revealed that all identified genes were enriched in ubiquitin-mediated proteolysis (ko04120) (Supplementary Fig. S6A), including E1 (Ubiquitin-activating enzyme), E2 (Ubiquitin-conjugating enzyme), and E3 (Ubiquitin ligase) genes (Supplementary Fig. S6B), implying that ubiquitination might be involved in *DlERF6*-mediated longan thermotolerance.

In this study, a decrease in the global ubiquitination level was detected in the *DlERF6*-OE roots compared with the WT roots under heat stress (Supplementary Fig. S7A). We further combined label-free immunoaffinity enrichment (antibody: PTM1104, PTM Biolabs) and high-resolution mass spectrometry to quantify protein ubiquitination in longan *DlERF6*-OE and WT roots under heat stress. The results showed that 2,754 sites (56.1%) in 1,488 proteins (57.3%) were downregulated and 2,155 sites (43.9%) in 1,110 proteins (42.7%) were upregulated (Supplementary Fig. S7B). As shown in Supplementary Fig. S7C, subcellular localization of the differentially ubiquitinated proteins were mainly localized in the cytoplasm (739, 34.87%), indicating that ubiquitination might play a crucial role in the cytoplasm during DlERF6-mediated heat stress response, by degrading the misfolded proteins (Zhang et al. 2021).

To demonstrate the functional differences between differentially ubiquitinated proteins, GO enrichment analysis was performed (Supplementary Fig. S7, D and E). In this study, we found that membrane protein, proteasome and cell wall were strongly enriched among the lysine ubiquitination (Kub) proteins with decreased ubiquitination, which may contribute to cell survival and repair under heat stress. In the biological process category, 28 terms were enriched in the differentially ubiquitinated proteins, implying that DlERF6 may be widely involved in ubiquitin-mediated biological processes under heat stress. In the molecular function analysis, sucrose synthase activity and oxidoreductase activity, proteins with binding activity were mainly enriched among downregulated ubiquitinated proteins. KEGG enrichment analysis suggested the protein-processing pathways in the aminoacyl biosynthesis, ascorbate and aldarate metabolism, glycometabolism, glutathione metabolism, pentose phosphate pathway, and flavonoid biosynthesis were enriched among upregulated ubiquitinated proteins (Supplementary Fig. S7F). The downregulated ubiquitinated proteins were enriched in pathways involving proteasome, starch and sucrose metabolism, vesicular transport, cutin, suberine and wax biosynthesis, and terpenoid backbone biosynthesis (Supplementary Fig. S7G).

Heat stress causes proteolysis *via* the ubiquitin–proteasome system (Wei et al. 2023). Under temperature stress, the main role of HSPs is to act as molecular chaperones for other cellular proteins to protect cells from stress (Sadura et al. 2020). In this study, we identified a series of HSPs that were differentially ubiquitinated between the *DlERF6*-OE and WT roots under heat stress (Supplementary Fig. S8). Moreover, heat stress increases the activity of antioxidant enzymes and protein synthesis, folding, and transport (Alcantud et al. 2023; Sun et al. 2023). In this study, antioxidant enzymes, including SOD, POD, CAT, and GST, were differentially ubiquitinated between *DlERF6*-OE and WT roots under heat stress (Supplementary Fig. S9). Flavonoids and plant cell wall proteins play important roles in heat stress perception, signaling, and recovery to maintain cellular homeostasis stability (Leal et al. 2022; Gao et al. 2023; Zhu et al. 2023). In this study, we revealed that numerous enzymes related to flavonoid and lignin biosynthesis, including flavanone 3-hydroxylase (F3H), chalcone synthase (CHS), chalcone isomerase (CHI), caffeoyl CoA O-methyltransferases (CCoAOMTs), phenylalanine ammonia-lyase (PAL), 4-coumarate coenzyme A ligase (4CL), and cinnamoyl-CoA reductase (CCR) proteins, were differentially ubiquitinated between the *DlERF6*-OE and WT lines under heat stress (Supplementary Fig. S10). We further compared the differentially ubiquitinated proteins with the proteome and transcriptome results. The results showed a weak positive association was observed between protein and mRNA; the mRNA level is not always conveyed to the final product protein. A weak correlation was observed between protein and mRNA levels, which may be attributed to protein stability and ubiquitination.

These results indicate that ubiquitination *via* DlERF6-mediated was related to primary metabolism, antioxidant substance, and secondary metabolites under heat stress in longan.

### DlERF6 directly represses *DlGH3.5* expression

**Figure 4.**
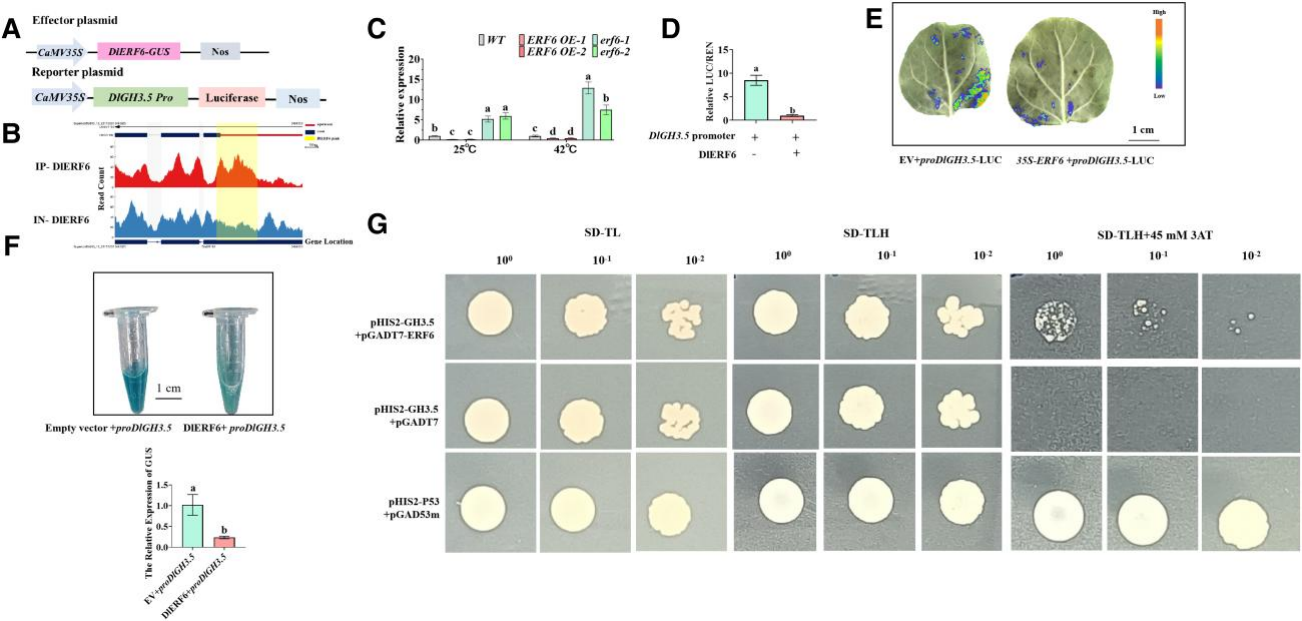
DlERF6 directly binds to *DlGH3.5*. **A)** Schematic diagrams of effector DlERF6 and reporter *DlGH3.5* vectors. **B)** The INTEGRATIVE GENOMICS VIEWER (IGV) image of DlERF6 binding to *DlGH3.5* promoter by DAP-seq. **C)** RT-qPCR analysis of *DlGH3.5* expression in wild-type (WT), *DlERF6* transgenic roots. One-way ANOVA was performed. Error bars represent the Sd of mean values (*n* = 3), and significant differences (*P* < 0.05) between groups are indicated by “a,” “b,” and “c.” **D, E)** Dual-luciferase system for the detection of DlERF6 targeting *DlGH3.5*. Images were digitally extracted for comparison. LUC, firefly luciferase activity; REN, Renilaluciferase. Student's *t*-test was performed. Error bars represent the Sd of mean values (*n* = 3), and significant differences (*P* < 0.05) between groups are indicated by “a,” “b,” and “c.” **F)** GUS staining assay and RT-qPCR showing that DlERF6 can inhibit *proDlGH3.5* expression. Images were digitally extracted for comparison. Student's *t*-test was performed. Error bars represent the Sd of mean values (*n* = 3), and significant differences (*P* < 0.05) between groups are indicated by “a”, “b” and “c”. **G)** Yeast one-hybrid analysis of the interaction between DlERF6 and *DlGH3.5* promoter.

We further examined the candidate target genes of DlERF6 in auxin signaling. ERF TFs can directly bind to the GCC-box motif in gene promoters to regulate their transcription (Liu et al. 2023). Taken together, the results of the DAP-seq and RNA-seq analyses revealed that DlERF6 may regulate the transcript level of *DlGH3.5* (*Dlo027192*) by directly binding to its promoter (GCC-box). The DAP-seq peaks in the promoter region of *DlGH3.5* contain the DlERF6 binding site (Fig. 4B). Consistent with these findings, the RT-qPCR results verified that the expression of *DlGH3.5* decreased in the *DlERF6*-OE roots but increased in the *DlERF6*-RNAi roots under both the control and heat treatments (Fig. 4C, Supplementary Table S10). To verify whether DlERF6 directly targeted the promoter sequence of *DlGH3.5*, we introduced the *DlGH3.5* promoter fragment into dual-luciferase systems (Fig. 4, A, D and E, Supplementary Table S11). Similarly, the GUS staining assay showed that longan callus coexpressing *DlERF6* and *DlGH3.5* promoter had lower GUS activity than callus expressing *DlGH3.5* promoter (Fig. 4F). Yeast one-hybrid analysis further indicated that DlERF6 could specifically bind to the GCC-box in the *DlGH3.5* promoter in vitro (Fig. 4G). These results suggest that DlERF6 can directly repress the transcription of *DlGH3.5*.

### *DlGH3.5* negatively regulates longan hairy root growth

**Figure 5.**
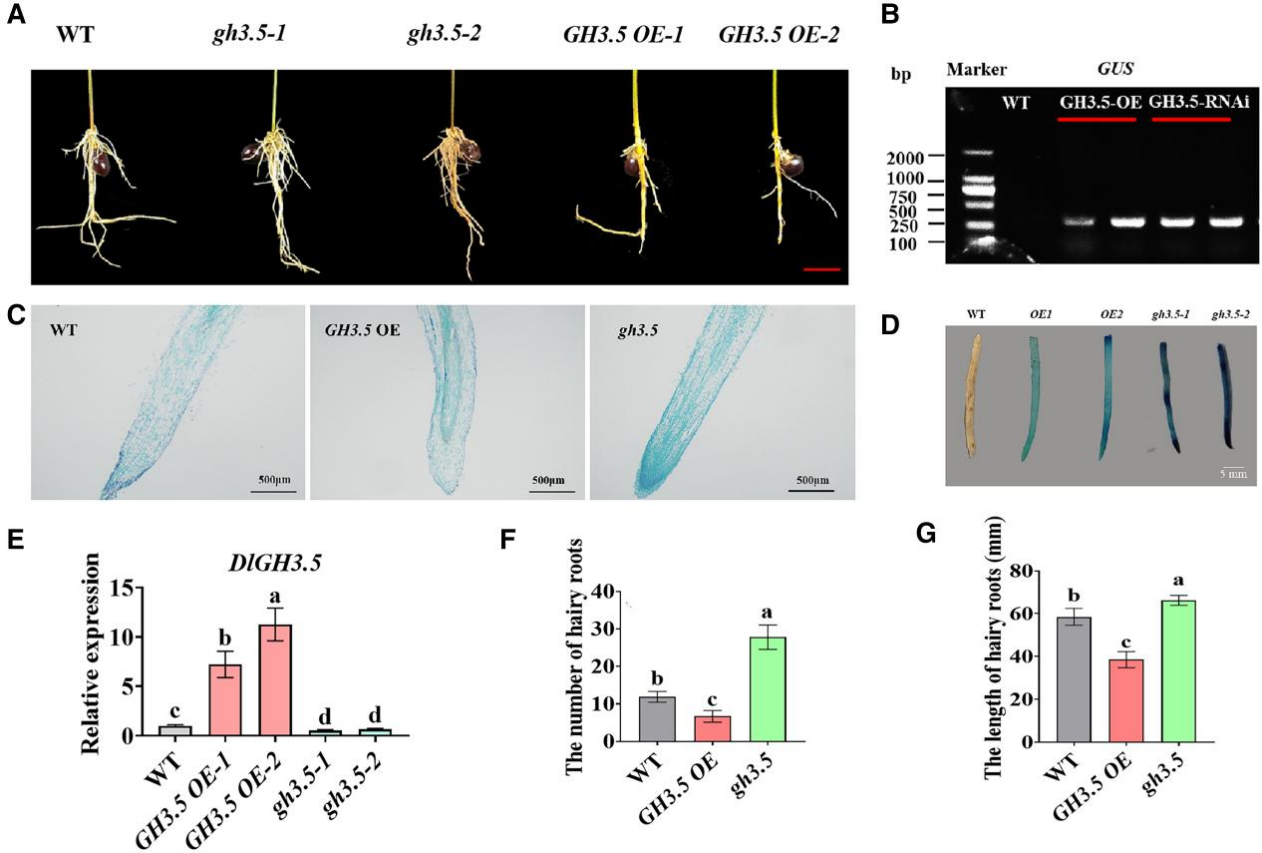
Determination of *DlGH3.5* transgenic roots. **A)** The phenotype of wild-type (WT), *DlGH3.5* transgenic roots. Bars = 5 cm. Images were digitally extracted for comparison. **B)** PCR analysis for GUS in independent wild-type (WT), *DlGH3.5* transgenic hairy roots. **C)** Longitudinal section of root tip region of hairy roots of WT, *DlGH3.5* transgenic lines. Roots were collected, in a paraffin section, and observed with 0.5% toluidine blue staining. **D)** GUS staining of the WT, *DlGH3.5* transgenic roots. Images were digitally extracted for comparison. **E)** RT-qPCR analysis of *DlGH3.5* in wild-type (WT)*, DlGH3.5* transgenic roots. OE 1,2, different *DlGH3.5*-overexpressing root lines. RNAi (*gh3.5-1*, *gh3.5-2*), different *DlGH3.5*-RNAi root lines. One-way ANOVA was performed. Error bars represent the Sd of mean values (*n* = 3), and significant differences (*P* < 0.05) between groups are indicated by “a,” “b,” and “c”. **F)** The statistical results of the hairy root numbers of wild-type (WT), *DlGH3.5* transgenic lines. One-way ANOVA was performed. Error bars represent the Sd of mean values (*n* = 10), and significant differences (*P* < 0.05) between groups are indicated by “a,” “b,” and “c.” **G)** The statistical results of the hairy root length of WT, *DlGH3.5* transgenic lines. One-way ANOVA was performed. Error bars represent the Sd of mean values (*n* = 10), and significant differences (*P* < 0.05) between groups are indicated by “a”, “b” and “c”.

To investigate the function of *DlGH3.5* in longan, *DlGH3.5-*OE and RNAi lines were obtained through hairy root transformation (Fig. 5A). The GUS staining and PCR results confirmed that the *DlGH3.5*-OE and RNAi fusion vectors had been transferred into longan roots and successfully induced the hairy roots, respectively (Fig. 5, B and D). The RT–qPCR results suggested that the expression level of *DlGH3.5* in *DlGH3.5-*OE roots was much greater than that in WT roots and that the level of *DlGH3.5* expression in RNAi roots was lower than that in WT plant roots (Fig. 5E, Supplementary Table S12). Morphologically, the number and length of hairy roots were lower in the *DlGH3.5*-OE lines than in the WT, whereas *DlGH3.5*-RNAi hairy roots were more abundant and grew faster (Fig. 5, F and G, Supplementary Tables S13 and S14). The cells in the root tip meristematic area of *DlGH3.5*-OE plants were arranged incompactly, and the shape was irregular. In contrast, the meristematic area of *DlGH3.5* RNAi cells was tighter and more regular, and the cell volume was smaller than that in WT roots (Fig. 5C). These results indicate that *DlGH3.5* negatively regulates longan hairy root growth.

### Overexpression of *DlGH3.5* confers heat sensitivity in longan by modulating auxin and ROS homeostasis

**Figure 6.**
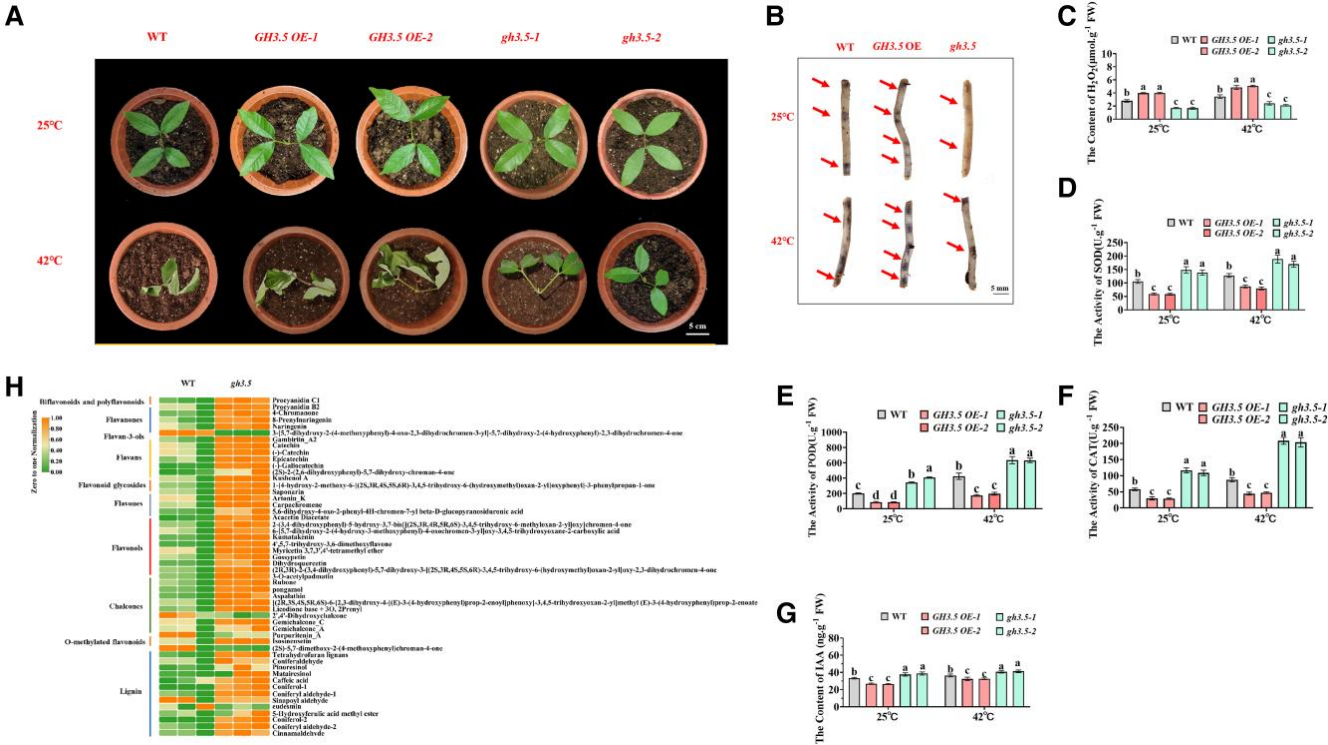
Overexpression of *DlGH3.5* reduces longan resistance to heat stress. **A)** Phenotype of plants wild-type (WT), overexpressing (OE), and RNAi *DlGH3.5* in the roots under heat treatment. Scale bar = 5 cm. Images were digitally extracted for comparison. **B)** Nitroblue tetrazolium (NBT) was used to detect the accumulation of superoxide and hydrogen peroxide (H_2_O_2_) in wild-type (WT), *DlGH3.5* transgenic roots. Images were digitally extracted for comparison. **C)** The H_2_O_2_ content of wild-type (WT), *DlGH3.5* transgenic roots under normal and heat treatment. FW: Fresh weight. One-way ANOVA was performed. Error bars represent the Sd of mean values (*n* = 3), and significant differences (*P* < 0.05) between groups are indicated by “a,” “b,” and “c.” **D–F)** The SOD, POD, and CAT activities of wild-type (WT), *DlGH3.5* transgenic roots under normal and heat treatment. FW: Fresh weight. One-way ANOVA was performed. Error bars represent the Sd of mean values (*n* = 3), and significant differences (*P* < 0.05) between groups are indicated by “a,” “b,” and “c.” **G)** The IAA (Indole-3-Acetic Acid) content of wild-type (WT), *DlGH3.5* transgenic roots under normal and heat treatment. FW: Fresh weight. One-way ANOVA was performed. Error bars represent the Sd of mean values (*n* = 3), and significant differences (*P* < 0.05) between groups are indicated by “a,” “b,” and “c.” **H)** Heatmaps of metabolites related to flavonoid and lignin synthesis in *DlGH3.5* RNAi roots in response to heat stress.

Furthermore, we investigated the heat tolerance of *DlGH3.5* transgenic lines. Two lines *DlGH3.5*-OE1 and *DlGH3.5*-OE2 with high expression levels of *DlGH3.5* and two RNAi lines (*gh3.5-1* and *gh3.5-2*) were obtained and selected for further analysis. All the plants grew well under normal conditions and presented similar phenotypes. However, after 3d of heat stress, the growth of the *DlGH3.5*-RNAi lines was greater than that of the WT plants. However, the *DlGH3.5*-OE lines were more severely damaged, indicating that *DlGH3.5* overexpression inhibited the growth of longan under heat stress (Fig. 6A). NBT staining revealed that, compared with WT roots, *DlGH3.5*-OE lines roots accumulated greater levels of ROS under normal and heat stress conditions. Conversely, the RNAi lines presented the least amount of accumulated ROS (Fig. 6B).

After heat stress, the H_2_O_2_ content increased in all the plants. The H_2_O_2_ content was evidently greater in the OE roots and leaves under heat stress conditions than in the WT roots and leaves (Fig. 6C, Supplementary Fig. S11A). The *DlGH3.5*-OE roots and WT roots contained 4.54∼5.14 *μ*mol/g and 3.16∼3.72 *μ*mol/g H_2_O_2_, respectively, whereas the RNAi roots contained only 2.00∼2.68 *μ*mol/g H_2_O_2_ under heat stress (Supplementary Table S15). SOD, POD, and CAT activities were lower in *DlGH3.5*-OE roots and leaves than that in WT (Fig. 6, D to F, Supplementary Fig. S11, B to D) under heat conditions. In contrast, the *DlGH3.5*-RNAi lines presented greater ROS scavenging ability in WT plants, and the *gh3.5-1* and *gh3.5-2* lines presented lower H_2_O_2_ contents than that in WT plants but greater antioxidant enzyme activities than that in WT plants (Fig. 6, D to F, Supplementary Fig. S11, B to D). These results suggested that *DlGH3.5* overexpression reduced the ability of longan to effectively eliminate ROS, consequently reducing heat tolerance in longan.

We further determined the IAA content of *DlGH3.5*-OE and RNAi roots under control and heat stress conditions. Under both conditions, compared with WT, *DlGH3.5* overexpression reduced the content of IAA, and more IAA accumulated in *DlGH3.5*-RNAi roots (Fig. 6G, Supplementary Table S15). The resistance of longan to heat stress mediated by *DlGH3.5* may be achieved by regulating ROS and auxin homeostasis.

To further verify whether flavonoid and lignin metabolic pathways are involved in *DlGH3.5-*mediated heat stress responses in longan, we next measured the contents of flavonoid and lignin metabolites in *DlGH3.5*-RNAi and WT roots under heat stress. As shown in Fig. 6H, most flavonoid and lignin metabolites were more abundant in *DlGH3.5*-RNAi lines under heat stress. Thirty-five flavonoid metabolites were increased, and four flavonoid metabolites were decreased in the *DlGH3.5*-RNAi roots compared with the WT roots, the increased flavonoid metabolites. Twelve lignin metabolites were increased, and 3 were decreased in the *DlGH3.5*-RNAi roots compared with the WT roots. These results suggested that *DlGH3.5* can negatively regulate flavonoid and lignin biosynthesis in longan roots under heat stress. In summary, these results indicate that *DlGH3.5* is a negative regulator of heat stress tolerance in longan.

## Discussion

Heat stress affects normal fruit tree growth and development and reduces quality and yield. Therefore, revealing the molecular regulatory mechanisms involved in the response to heat stress will provide insights for fruit tree breeding in the future. As the roots of a plant are responsible for water and nutrient uptake from the soil, the survival rate of plants under abiotic stress conditions largely depends on root development and modulation (Grossman and Rice 2012; Suralta et al. 2018; Seo et al. 2020). For example, root-specific overexpression of *OsNAC5*, *OsNAC6*, OsNAC9, and *OsNAC10* increases the root diameter and thereby enhances stress tolerance (Redillas et al. 2012; Jeong et al. 2013, 2010; Lee et al. 2017). The generation of *GmMYB118*-OE soybean plants *via A*. *rhizogenes*-mediated transformation of hairy roots increases plant drought and salt tolerance (Du et al. 2018). Previous studies indicate that ERF family genes play crucial roles in plant growth, development, and stress response by regulating the transcription of downstream target genes (Licausi et al. 2013; Abiri et al. 2017; Phukan et al. 2017). ERF115, ERF114, and ERF109 are involved in quiescent central cell division in Arabidopsis roots by activating the transcription of *PSK5* and *PSK2* (Kong et al. 2018). In this study, *DlERF6* was expressed predominantly in the roots of longan and was upregulated by IAA treatment (Supplementary Fig. S1, A and C). As shown in Fig. 1, F and G, overexpression of *DlERF6* increased the number and length of longan hairy roots. In contrast, the number and elongation of hairy roots in *DlERF6-*RNAi lines were decreased compared with WT plants. In addition, the meristematic area of the *DlERF6*-OE root tips was larger, and the cells were arrayed more neatly and closely (Fig. 1C). These results showed that *DlERF6* plays a positive role in the regulation of longan root architecture, length, and number.

Auxin and ROS homeostasis play crucial roles in the initiation and promotion of roots (Fukaki and Tasaka 2009; Neogy et al. 2019). In this study, the overexpression of *DlERF6* markedly increased the free IAA content, whereas the inhibition of *DlERF6* decreased the IAA content in longan roots (Fig. 3A). Compared with WT roots, *DlERF6-*OE roots presented a lower H_2_O_2_ content (Fig. 2C). In rice, *OsGH3-2* and *OsGH3*-*13* reduce free IAA levels and root numbers (Zhang et al. 2009; Du et al. 2012). Moreover, *GH3.5* and *GH3.6* reduce the apple IAA content and inhibit adventitious root growth (Zhao et al. 2020; Jiang et al. 2022). *CsGH3.2* and *CsGH3.3* in rice inhibit the formation of adventitious roots (Chen et al. 2022). *CsGH3.4* suppresses the development of adventitious roots by regulating auxin levels in tea plants (Wang et al. 2024a, 2024b). *AtGH3-6* inhibits shoot elongation and lateral root formation (Nakazawa et al. 2001). Shorter primary roots and fewer lateral roots are observed in *AtGH3-2* mutant lines (Takase et al. 2004). *AtGH3.3*, *AtGH3.5,* and *AtGH3.6* positively regulate adventitious root growth (Gutierrez et al. 2012). In this study, DAP-Seq, dual-luciferase, GUS-reporter, and yeast one-hybrid assays confirmed that DlERF6 directly binds to the promoter of *DlGH3.5* and negatively regulates its expression (Fig. 4). A decrease in root number and length was detected in *DlGH3.5*-OE lines, and an increase in root abundance was detected in *DlGH3.5*-RNAi lines (Fig. 5A). Additionally, the inhibition of *DlGH3.5* in longan led to a decrease in H_2_O_2_ content and an increase in free IAA content (Fig. 6, C and G). These findings are consistent with the role of *DlERF6* in root development, implying that DlERF6 promotes longan root development by inhibiting the expression of *DlGH3.5* to regulate IAA and ROS homeostasis.

The expression of ERFs increases in plants under cold stress (Dey and Corina Vlot 2015). In rice, *OsSERF1* is phosphorylated and activated in response to salinity stress (Schmidt et al. 2013). ERF98 enhances salt tolerance in Arabidopsis by activating ascorbic acid biosynthesis genes (Zhang et al. 2012). Moreover, ERF15 positively regulates Populus drought resistance by interacting with jasmonic acid-mediated signaling (Kong et al. 2023). *OsERF5* promotes resistance to drought stress in rice (Pan et al. 2012). In tomatoes, *TERF1* enhances rice drought and salt tolerance by promoting proline biosynthesis (Gao et al. 2007). In this study, we found that *DlERF6* was induced by heat treatment (Supplementary Fig. S1B), with *DlERF6* overexpression enhancing tolerance to heat stress in longan (Fig. 2).

Under heat stress, plants produce excessive ROS, which can cause oxidative stress and cell death (Mittler et al. 2004; Li et al. 2023). Therefore, enhancing the ROS scavenging ability of plants is crucial for plant survival under heat stress. In Arabidopsis, ERF71 and ERF73 are involved in the regulation of plant ROS homeostasis (Yao et al. 2017). *ZmEREB20* improves maize salt tolerance by regulating ROS scavenging and hormone signaling (Fu et al. 2021). SOD, POD, CAT, and GST are the major antioxidant enzymes that scavenge ROS (Choudhury et al. 2017). In this study, heat stress clearly altered the morphology and physiology of the WT, *DlERF6*-OE, and *DlERF6*-RNAi lines. The plants with *DlERF6*-OE roots presented greater heat resistance than WT plants. However, the reduced expression of *DlERF6* decreased longan heat stress tolerance. Lower H_2_O_2_ accumulation and higher SOD, POD, CAT, and GST activities were detected in the plants with *DlERF6-*OE roots under heat stress, indicating that these plants had a more efficient antioxidant system (Fig. 2, B to F, Supplementary Fig. S2). The greater H_2_O_2_ accumulation in the *DlERF6*-RNAi lines resulted in severe cellular damage, which may have reduced their heat tolerance. Additionally, plants can also produce nonenzymatic antioxidants to mitigate excessive ROS damage (Muhlemann et al. 2018). For example, flavonoids can effectively scavenge free radicals in sweet potato and onion leaves (Chu et al. 2000). Flavonoid compounds have been reported to play protective roles against abiotic stresses, such as low temperatures, drought and high salinity conditions, through the detoxification of ROS (Agati et al. 2013; Šamec et al. 2021; Gao et al. 2023). Lignin, a polymer composed of phenylpropanoid compounds, is often reported to be involved in responses to abiotic stresses (Rogers and Campbel 2004). As shown in Supplementary Fig. S4D, compared with those in the WT roots, the levels of most flavonoids and lignin metabolites were increased in the *DlERF6*-OE roots under heat stress. These findings suggest that *DlERF6* is involved in heat stress through the regulation of flavonoid biosynthesis. Based on the RNA-seq analysis, we further identified the ROS scavenging-related genes with elevated expression, including antioxidant enzyme genes (Supplementary Fig. S3, C and D), and key genes in flavonoids and lignin biosynthesis in *DlERF6*-OE roots compared with the WT roots under heat stress (Supplementary Fig. S4A). These genes may contribute to increasing the levels of antioxidant enzymes and metabolites to increase the ability of *DlERF6*-OE lines to detoxify ROS under heat stress.

Auxin signaling-related genes greatly enhance abiotic stress tolerance by increasing the ability of plants to scavenge ROS (Naser and Shani 2016; Bielach et al. 2017). *YUCCA6* enhances potato or poplar drought stress tolerance by promoting auxin production and reducing ROS levels (Kim et al. 2013; Ke et al. 2015). In this study, we found that *DlERF6* overexpression increased the accumulation of free IAA and decreased the content of IAA-amino acid conjugates under heat stress (Fig. 3, A and B). We speculated that increased auxin accumulation may provide feedback to increase ROS scavenging efficiency and modulate heat stress tolerance in *DlERF6*-OE roots. A relatively high level of H_2_O_2_ may further inhibit IAA synthesis in *DlERF6-*RNAi roots, which decreases longan heat stress tolerance (Jia et al. 2016). According to RNA-seq results, we found that the expression of auxin biosynthesis genes was higher and the expression of auxin conjugation and degradation genes were lower in *DlERF6*-OE roots compared with the WT roots under heat stress (Fig. 3C), indicating that *DlERF6* positively regulated IAA synthesis in longan under heat stress. Higher antioxidant enzyme activities and metabolite and IAA contents resulted in more efficient ROS scavenging in longan *DlERF6-*OE lines under heat stress. It can be assumed that *DlERF6* can improve the ROS and IAA balance in longan, which can directly or indirectly improve the plant's heat tolerance.

In Sorghum, *GH3* genes are induced in response to salt and drought stress (Wang et al. 2010). In Medicago, *MtGH3*-*7* is upregulated in the roots by drought stress (Singh et al. 2015). *OsGH3*-*2* negatively regulates drought tolerance by impairing ROS scavenging (Du et al. 2012). In potatoes, *StGH3.3* increases drought tolerance by enhancing ROS scavenging (Yao et al. 2023). In cotton, *GH3.5* positively regulates drought tolerance, with a greater ability to scavenge ROS (Kirungu et al. 2019). *MdGH3.6* negatively regulates ROS scavenging in apple plants by reducing the levels of antioxidant enzymes, phenylpropanoid metabolites, and flavonoids under drought stress (Jiang et al. 2022). These results indicate multifarious functions of GH3 family members in the plant response to abiotic stresses. In this study, we found that overexpression of *DlGH3.5* decreased heat stress tolerance in longan. H_2_O_2_ accumulation was much greater in the *DlGH3.5-*OE seedlings than in the WT seedlings under normal and heat stress conditions. The activities of SOD, POD, and CAT were much lower in the *DlGH3.5*-OE plants than in the WT plants under heat stress (Fig. 6, D to F, Supplementary Fig. S11). In contrast, the H_2_O_2_ level was lower in the *DlGH3.5* RNAi plants than in the WT plants under heat stress. Accordingly, the activities of SOD, POD and CAT were greater in *DlGH3.5* RNAi plants than in WT plants in response to heat stress. In addition, compared with those in WT roots, the levels of most flavonoids and lignin metabolites were increased in *DlGH3.5* RNAi roots under heat stress. The increased levels of IAA, flavonoids, and lignin metabolites, as well as antioxidant enzyme activities in the *DlGH3.5* RNAi roots, may have induced the plant's heat resistance.

**Figure 7.**
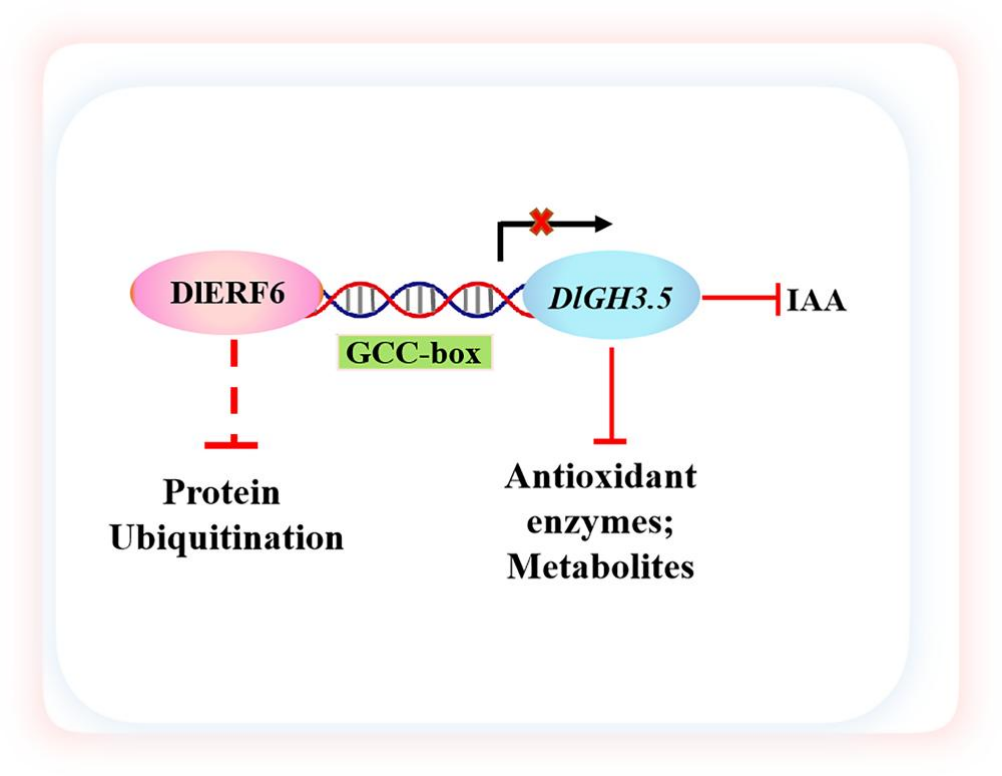
Proposed working model of DlERF6–*DlGH3.5*. DlERF6 directly inhibits the expression of *DlGH3.5* by binding to a GCC-box motif in its promoter. DlERF6 positively regulates the Indole-3-Acetic Acid (IAA) accumulation and enhances the reactive oxygen species (ROS) scavenging ability. Arrows and bar ends indicate activation and repression effects, respectively. Dashed lines suggest potential regulatory. Solid lines indicate direct regulatory relationships.

In summary, we identified DlERF6 as a positive regulator of hairy root growth and heat stress tolerance in longan (Fig. 7). Healthy root systems are essential for nutrient and water uptake, thereby enhancing overall plant resilience. In this study, increased root mass and well-developed hairy roots may have facilitated improved survival and growth under heat stress. Compared with WT plants, the plants with *DlERF6*-OE roots were more tolerant to heat stress as a result of auxin and ROS homeostasis restoration, which involved increases in the activities of antioxidant enzymes and the levels of the antioxidant metabolites flavonoids and lignin. In addition, under heat stress treatment, DlERF6-mediated ubiquitination is likely achieved by regulating protein activity, transport, and interactions. *DlERF6* was activated under heat stress, and *DlGH3.5* was a direct target of DlERF6 and a negative regulator of heat resistance in longan. Consequently, *DlERF6* functions as a positive regulator of root growth and heat tolerance by negatively regulating the expression of *DlGH3.5*.

## Materials and methods

### Plant materials and treatments

The *Dimocarpus longan* var “*ShiXia*” (“*SX*”) seeds were germinated for 2 wk in pots containing vermiculite. The longan seedlings were grown in a greenhouse at 25 ℃ and 75% relative humidity under long-day conditions (16-h light/8-h dark photoperiod) with a light intensity of about 75 mmol photons m^−2^s^−1^. For heat stress treatment, the one-month-old longan seedlings were placed in a 42 ℃ chamber for heat treatment, for 24 and 48 h. For IAA treatment, the seedlings were subjected to 1 mg·L^−1^ IAA solution for 24 and 48 h. Ten plants were used for each treatment. All harvested seedlings were submerged immediately in liquid nitrogen and stored at −80 ℃ for RNA extraction.

### Phylogenetic tree construction and sequence alignment

The full-length amino acid sequences (*Dimocarpus longan*, *Arabidopsis thaliana*, *Gossypium hirsutum*, and *Vitis vinifera*) were downloaded from the NCBI (https://www.ncbi.nlm.nih.gov/). The phylogenetic trees were constructed in MEGA6.0 based on the neighbor-joining method as previously reported (Kumar et al. 2018), and was visualized using the iTOL tool (https://itol.embl.de/). Sequence alignment was constructed in DNAMAN (version 7.0; Lynnon Biosoft, Quebec, Canada).

### Subcellular localization of DlERF6

The *DlERF6* coding sequence was inserted into the pCAMBIA-1302-35S expression vector to express the *DlERF6*-green fluorescent protein (GFP) fusion protein. Primers are in Supplementary Table S16. The onion inner epidermis was infected by *Agrobacterium* (GV3101) *tumefaciens* mediated. After 48 ∼ 72 h of culture, the inner epidermis was immersed in a 4, 6-diamidino-2-phenylindole (DAPI, identify the nucleus) staining solution containing 1 *μ*g ml^−1^ DAPI for 10 min; the GFP signaling was observed and imaged using confocal microscopy. Fluorescence was detected using the Olympus FV1200 confocal laser microscope (Tokyo, Japan). DAPI was excited at 405 nm (0.8% laser power) and detected at 450 to 490 nm. Green fluorescent protein was excited at 488 nm (3.6% laser power), and the emission bandwidth was 500 to 530 nm.

### Total RNA extraction and gene expression pattern analysis

Total RNA was extracted and the RT-qPCR was performed according to our previous methods (Zhang et al. 2023). Total RNA was extracted using the TransZol Up reagent kit (TIANGEN, Beijing, China) and treated with RNase-free DNase I (TIANGEN, Beijing, China) following the manufacturer's instructions. Complementary DNA was synthesized using first-strand cDNA using a PrimeScript RT Master Mix (Perfect Real Time) cDNA Synthesis Kit (TaKaRa, Japan). The RT-qPCR was performed using the LightCycler 480 platform (Roche Applied Sciences, Basel, SC). Each experiment was repeated 3 times. *UBIQUITIN* (*UBQ*) was used as an endogenous reference gene. The experimental results were shown from all 3 biological replicates using the 2^−△△Ct^ statistical method (Livak and Schmittgen 2001). The primer sequences are listed in Supplementary Table S16.

### *Agrobacterium Rhizogenes*-mediated transformation

Longan cultivar “*ShiXia*” (“*SX*”) was used for *Agrobacterium rhizogenes-*mediated transformation to construct longan hairy roots. The coding sequences (CDS) of *DlERF6* and *DlGH3.5* was cloned into vector pCAMBIA-1301-35S. For the *DlERF6* and *DlGH3.5* RNA interference (*DlERF6*-RNAi, *DlGH3.5*-RNAi) construct, a 312-bp *DlERF6* fragment and a 420-bp *DlGH3.5* fragment, and its antisense sequence was synthesized and inserted into pTCK303, respectively. The recombinant constructs and WT (K599 empty strain as the control) were transferred into *A. rhizogenes* strain K599 (Weidi Biotechnology, Shanghai, China). Longan transformation to induce transgenic hairy roots was performed as a previous method (Meng et al. 2019). Transgenic hairy roots were generated from the infection sites in about 3∼4 wks, and the original main roots were removed. GUS staining and PCR were used to verify the positive hairy roots. After identification, plants retained positive hairy roots for subsequent heat tolerance analysis. Afterward, the transgenic hairy root seedlings were subjected to 42 ℃ treatment for 3d. At least 30 seedlings per genotype were measured, and the heat stress assay was performed at least 3 times. The primers are in Supplementary Table S16.

### RNA-seq analysis

To analyze global gene expression changes in the *DlERF6* OE plants, the roots obtained from *DlERF6* OE and WT plants under heat stress conditions for 3d were used for RNA extraction. The total RNA was extracted for RNA sequencing using the TransZol Up reagent kit (TIANGEN, Beijing, China). Subsequent procedures were conducted on the Illumina NovaSeq 6000 platform (Biomarker Technologies Co., Ltd., Wuhan, China). The final size and quality of the libraries were assessed using the Qsep400 and Qubit Fluorometer (Life Technologies, Carlsbad, CA, USA). The RNA-seq data were submitted to the National Genomics Data Center (https://ngdc.cncb.ac.cn/bioproject/, China) with BioProject number PRJNA1102508. Heatmaps were constructed by Tbtools (Chen et al. 2020).

### Untargeted metabolomic analysis

The roots from the heat stress for 3d plants of WT, *DlERF6-OE,* and *DlGH3.5*-RNAi were harvested for the untargeted metabolomic analysis. Three biological replicates were performed. For LC-MS/MS analysis, the samples of the supernatants were transferred to UHPLC system (Vanquish, Thermo Fisher Scientific). The identification and quantification of the metabolites were performed with the assistance of Biotree Biotechnology (Shanghai, China). Heatmaps were constructed by Tbtools (Chen et al. 2020).

### Western blotting analysis

For the global levels of protein ubiquitination in longan *DlERF6*-OE, *DlERF6*-RNAi, and WT roots, total proteins were extracted and subjected to 12% SDS-PAGE. Ubiquitination detection was constructed by the primary anti-ubiquitin antibody (1:1,000 dilution, PTM-1106RM; Lot: RN010622, PTM Bio, Hangzhou, China). The secondary antibody was Thermo, Pierce, Goat anti-Rabbit IgG, (H + L), Peroxidase Conjugated, 31460, 1:10,000 dilution. Signals were collected by the Tanon 5200 Multi Imaging System (Shanghai, China) following the manufacturer's instructions.

### Ubiquitination analysis

The roots from the 3-day heat-stress-treated seedlings of WT and *DlERF6*-OE were harvested for the ubiquitinome profiling analysis. Three seedlings samples were mixed. The assay was performed as described by Zhu et al. (2020b). NanoElute UHPLC system (Bruker Daltonics) was used for LC-MS/MS analysis. Functional enrichment analysis for differentially expressed ubiquitinated proteins using Fisher's exact test (background: the identified protein). PSORTb software was applied to predict the identified proteins subcellular location.

### Dual-luciferase reporter assays in *Nicotiana benthamiana* leaves

The CDS of *DlERF6* was cloned into the pCAMBIA-1301-35S vector. Promoter sequences of *DlGH3.5* were cloned into the pGreenII 0800-LUC vector. The primers are listed in Supplementary Table S16. The fusion constructs were infected into *Nicotiana benthamiana* (Cao et al. 2024). The LUC fluorescence signal was determined using chemiluminescence imaging system (Gelview 6000Pro II, BLT, China), and firefly luciferase (LUC) and Renilla luciferase (REN) activity levels were detected using a dual-luciferase reporter gene assay kit (Thermo, Shanghai, China). The LUC activity was normalized to REN activity. Six biological replicates were performed.

### GUS analysis

The promoter of *DlGH3.5* was cloned into the pCAMBIA-GUS vector, and longan callus was co-transformed with 35S::DlERF6-GFP and *proDlGH3.5*-GUS using a previous method (Zhang et al. 2023). GUS staining solution (GT0391, HUYUEYANG, China) for 48 h at 37 ℃. Three biological replicates were performed for each treatment.

### Y1H assay

Inserts containing the ORFs of *DlERF6* were cloned into the *pGADT7* vector as the prey. Likewise, inserts containing the promoter of *DlGH3.5* were cloned into the *pHIS2* vector as the bait. The 3-AT was used to suppress the expression of *pHIS2*-*proDlGH3.5*. Yeast strain pairs: pGADT7-ERF6 + pHIS2-*GH3.5*, pGADT7 + pHIS2-*GH3.5*, pGAD53 m + pHIS2-p53 were obtained. The yeast strain Y187 was transformed with fusion vectors for next Y1H assays (Liu et al. 2022). Protein-DNA interaction was detected according to the manufacturer's protocol (45 mm 3-AT; Clontech, CA, USA).

### DAP-Seq assay

DAP-Seq assay was performed according to the previous study by Bartlett et al. (2017) with minor modifications. The CD sequence of *DlERF6* was cloned into the pFN19 K (HaloTag) T7SP6 Flexi vector (Pro mega G184A). Illumina HiSeq TM 4000 by Gene Denovo Biotechnology Co. was applied to detect the eluted DNA fragments (Guangzhou, China). The clean reads were aligned by Bowtie2 (version: 2.2.5) against the longan genome (Chen et al. 2023). The identified peaks were called using MACS2. MEME software was used for motif analysis. The peak-related gene annotations were finished by the R package (Yu et al. 2015). The enrichment analysis of the genes associated with different peaks was identified (Hutin et al. 2023).

### Measurement of physiological indicators

The H_2_O_2_ content was determined using the content determination kits (Mlbio, Shanghai, China). The activities of GST, CAT, POD, and SOD were determined using the corresponding assay kits (Comin Biotechnology, Suzhou, China). About 0.5 g fresh weight (FW) was used to detect IAA content, which was performed by the ELISA kit (Mlbio, Shanghai, China) following the manufacturer's instructions (Yang et al. 2001). IAA-aa were analyzed using he UHPLC separation which was carried out using ExionLC AD UHPLC System (SCIEX), equipped with a UPLC Kinetex C18 (2.1 mm × 100 mm, 2.6 *μ*m). The mobile phase A was 0.1% formic acid in water, and the mobile phase B was 0.1% formic acid in methanol. The column temperature was set at 25 ℃. The auto-sampler temperature was set at 4 ℃, and the injection volume was 2 *μ*L.

### Accession numbers

Sequence data from this article can be found in the National Centre for Biotechnology Information (NCBI) under accession number PRJNA792504.

## Supplementary Material

kiaf096_Supplementary_Data

## Data Availability

The data that support the findings of this study are available from the corresponding author upon reasonable request.

## Supplementary data

The following materials are available in the online version of this article.

**Supplementary Figure S1.** Expression analysis of DlERF6 and characterization of DlERF6.

**Supplementary Figure S2.** The reactive oxygen species (ROS) levels of Wild-Type (WT) and DlERF6 transgenic plant leaves under normal and heat stress treatment.

**Supplementary Figure S3.** RNA-seq analysis of *DlERF6* overexpression plants in response to heat stress.

**Supplementary Figure S4.** The flavonoid and lignin metabolic pathways are involved in *DlERF6*-mediated resistance to heat stress.

**Supplementary Figure S5.** Genome-wide analysis of DlERF6 binding sites and target genes using DAP-seq.

**Supplementary Figure S6.** Enrichment analysis of all target genes regulated by *DlERF6*.

**Supplementary Figure S7.** Overview of ubiquitylation in *DlERF6*-mediated resistance to heat stress.

**Supplementary Figure S8.** Comparative analysis of ubiquitination and expression patterns of HSP proteins in *DlERF6* Overexpression (OE) vs Wild-Type.

**Supplementary Figure S9.** Comparative analysis of ubiquitination and expression patterns of ROS-related proteins in DlERF6 overexpression (OE) vs wild-type (WT) root.

**Supplementary Figure S10.** Comparative analysis of ubiquitination and expression patterns of flavonoid and lignin biosynthesis-related Proteins in DlERF6 overexpression (OE) vs wild-type (WT) roots.

**Supplementary Figure S11.** Effects of heat stress on H2O2 content and antioxidant enzyme activities in wild-type (WT) and *DlGH3.5* transgenic plants.

**Supplementary Table S1.** Relative expression of *DlERF6* under heat stress and IAA treatment in longan roots.

**Supplementary Table S2.** Relative expression of *DlERF6* in *DlERF6* overexpression (OE) and *erf6* mutants.

**Supplementary Table S3.** Hairy root number in *DlERF6* overexpression (OE) and *DlERF6* RNAi lines.

**Supplementary Table S4.** Hairy root length in *DlERF6* overexpression (OE) and *DlERF6* RNAi lines.

**Supplementary Table S5.** Quantification of physiological indicators in *DlERF6* overexpression (OE) and *DlERF6* RNAi roots.

**Supplementary Table S6.** RNA-seq analysis of *DlERF6* overexpression roots in response to heat stress.

**Supplementary Table S7.** Expression analysis of transcription factor-encoding genes in *DlERF6* overexpression roots in response to heat stress.

**Supplementary Table S8.** Metabolites related to flavonoid biosynthesis in *DlERF6* overexpression and *DlGH3.5* RNAi roots in response to heat stress.

**Supplementary Table S9.** Metabolites related to lignin biosynthesis in *DlERF6* overexpression and *DlGH3.5* RNAi roots in response to heat stress.

**Supplementary Table S10.** Relative expression of *DlGH3.5* in *DlERF6* overexpression and *DlERF6* RNAi roots.

**Supplementary Table S11.** Relative LUC/REN ratios.

**Supplementary Table S12.** Relative expression of *DlGH3.5* in *DlGH3.5* overexpression and *DlGH3.5* RNAi roots.

**Supplementary Table S13.** Hairy root number in *DlGH3.5* overexpression and *DlGH3.5* RNAi lines.

**Supplementary Table S14.** Hairy root length of *DlGH3.5* overexpression and *DlGH3.5* RNAi lines.

**Supplementary Table S15.** Quantification of physiological indicators in *DlGH3.5* overexpression and *DlGH3.5* RNAi roots.

**Supplementary Table S16.** List of primers used in this study.

## Dive Curated Terms

The following phenotypic, genotypic, and functional terms are of significance to the work described in this paper:

erf2 Gramene: AT5G47220
erf2 Araport: AT5G47220

DREB2A Gramene: AT5G05410
DREB2A Araport: AT5G05410

ERF1 Gramene: Acc25620
ERF1 Araport: Acc25620

TAA1 Gramene: At1g70560
TAA1 Araport: At1g70560

ELISA Gramene: enzyme linked immunosorbent assay
ELISA Araport: enzyme linked immunosorbent assay

YUCCA6 Gramene: AT5G25620
YUCCA6 Araport: AT5G25620

YUC8 Gramene: At4g28720
YUC8 Araport: At4g28720
