# Supplementary material for: The ETHYLENE RESPONSE FACTOR6-GRETCHEN HAGEN3.5 module regulates rooting and heat tolerance in Dimocarpus longan
Source: Plant Physiol. 2025 Mar 19;197(3):kiaf096. doi: 10.1093/plphys/kiaf096 (PMC11950727; doi:10.1093/plphys/kiaf096)
Supplement: kiaf096_Supplementary_Data [file kiaf096_supplementary_data.zip › Supplementary Data.pdf]

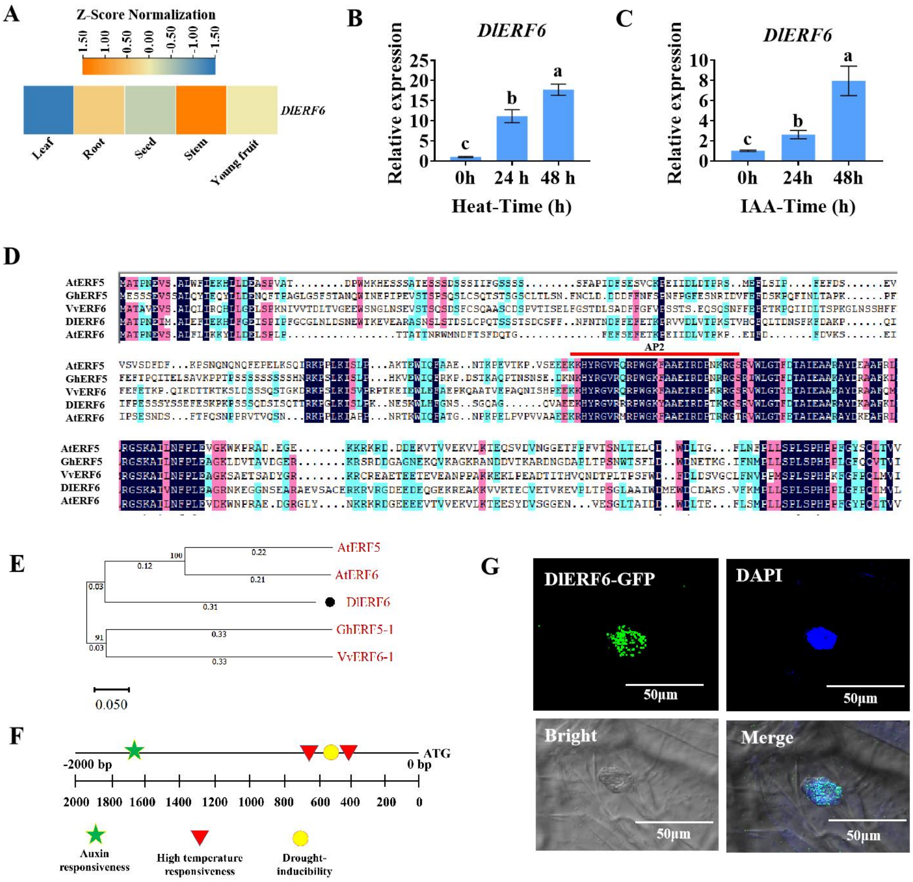

Figure S1 Expression analysis of *DlERF6* and characterization of DlERF6.

A Heatmap of *DlERF6* in longan different tissues. **B** Expression analysis of *DlERF6* under heat stress treatment in longan roots. Error bars represent the SD of mean values (n=3), and significant differences (P < 0.05) between groups are indicated by 'a','b' and 'c'. **C** Expression analysis of *DlERF6* under IAA treatment in longan roots. **D** Amino acid sequence alignment of DlERF6 with homologous protein; AP2 domain were marked using red line. The same or similar sequences are labeled with the same color background. **E** Phylogenetic tree of DlERF6 based on other four ERF proteins. Bootstrap values were based on 500 replicates. The 0.05 scales indicate substitution distance. **F** Prediction of cis-acting elements of the DlERF6 promoter. **G** The subcellular localization of DlERF6. DlERF6-GFP (Green fluorescent protein) was transiently expressed in onion epidermal cells. The GFP signal overlaps with the DAPI signal. Bar, 50 μm. One way ANOVA was performed. Error bars represent the SD of mean values (n=3), and significant differences (P < 0.05) between groups are indicated by 'a','b' and 'c'.

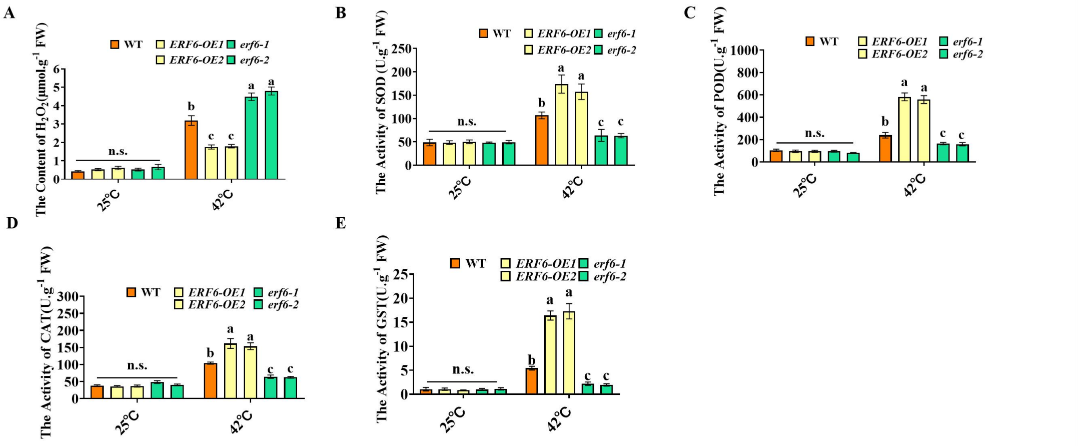

Figure S2 The reactive oxygen species (ROS) levels of Wild-Type (WT) and DlERF6 transgenic plants leaves under normal and heat stress treatment. A The H_2O_2 content in the leaves of Wild-Type (WT), *DlERF6* overexpression lines and *erf6* mutants under normal and heat stress conditions. **B-E** The SOD, POD, CAT and GST activities of Wild-Type (WT) and *DlERF6* transgenic plants leaves under normal and heat stress treatment. One way ANOVA was performed. Error bars represent the SD of mean values (n=3), and significant differences (P < 0.05) between groups are indicated by 'a','b' and 'c'.

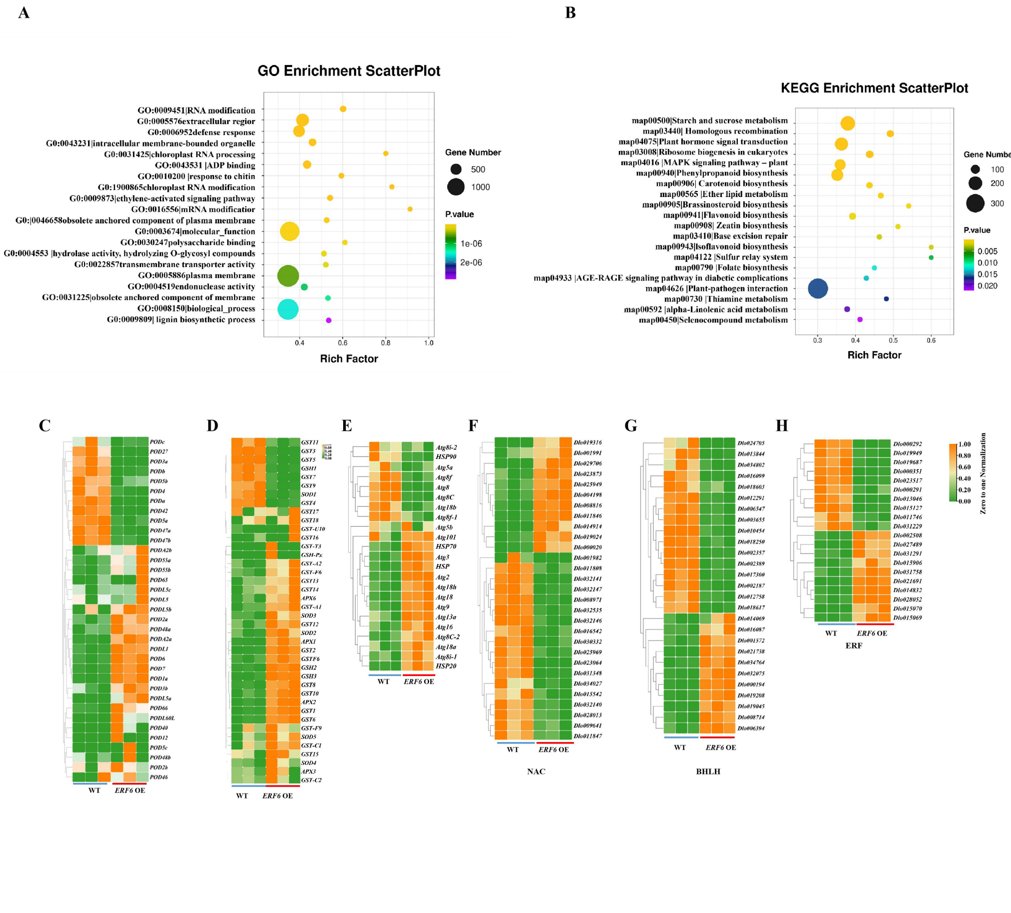

Figure S3 RNA-seq analysis of *DlERF6* overexpression plants in response to heat stress.

A GO enrichment of differentially expressed genes in *DlERF6* overexpression roots in response to heat stress. **B** KEGG enrichment of differentially expressed genes in *DlERF6* overexpression roots in response to heat stress. **C-D** Expression patterns of antioxidant metabolism genes in *DlERF6* overexpression roots in response to heat stress. **E** Expression patterns of autophagy pathway and heat shock proteins genes in *DlERF6* overexpression roots in response to heat stress. **F-H** Expression patterns of NAC, BHLH and ERF transcription factor genes in *DlERF6* overexpression roots in response to heat stress. Green color indicates low expression level whereas orange color indicates high expression level.

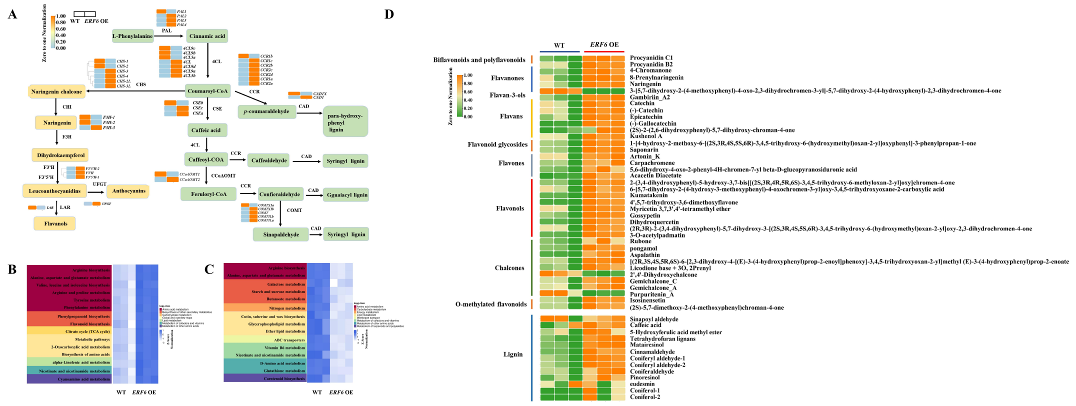

Figure S4 The flavonoid and lignin metabolic pathways are involved in DlERF6-mediated resistance to heat stress

A Expression of flavonoid and lignin metabolism genes in *DlERF6* overexpression (OE) roots in response to heat stress. **B, C** KEGG enrichment analysis of up- and down-regulated metabolites in *DlERF6* overexpression (OE) roots in response to heat stress, respectively. **D** Heatmaps of metabolites related to flavonoid and lignin synthesis in *DlERF6* overexpression (OE) roots in response to heat stress. Green color indicates low expression level whereas orange color indicates high expression level.

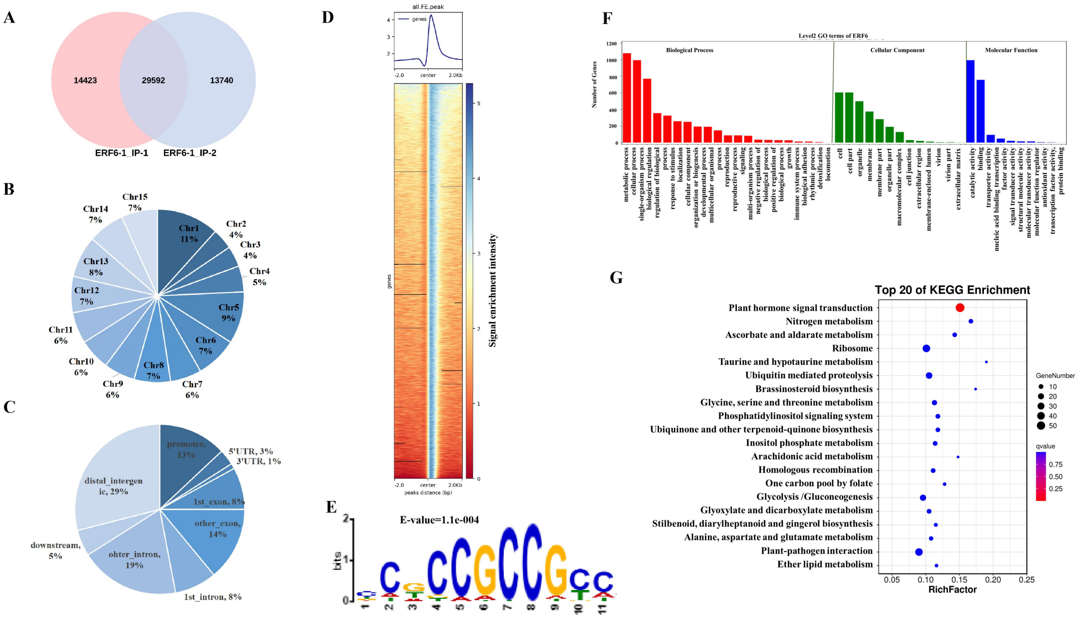

Figure S5 Genome-wide analysis of DlERF6 binding sites and target genes using DAP-seq

A Overlap of DlERF6 binding peaks in two technical replicates of DNA affinity purification sequencing (DAP-seq). **B** Distribution of peaks in chromosome of target genes. **C** Distribution of peaks in different regions of target genes. **D** Metaplots and Heatmap of DAP-seq signals. **E** Potential binding elements of DlERF6 identified by DAP-seq. **F** GO enrichment analysis of DlERF6 target genes with promoters binding site. **G** KEGG enrichment analysis of DlERF6 target genes with promoters binding sites.

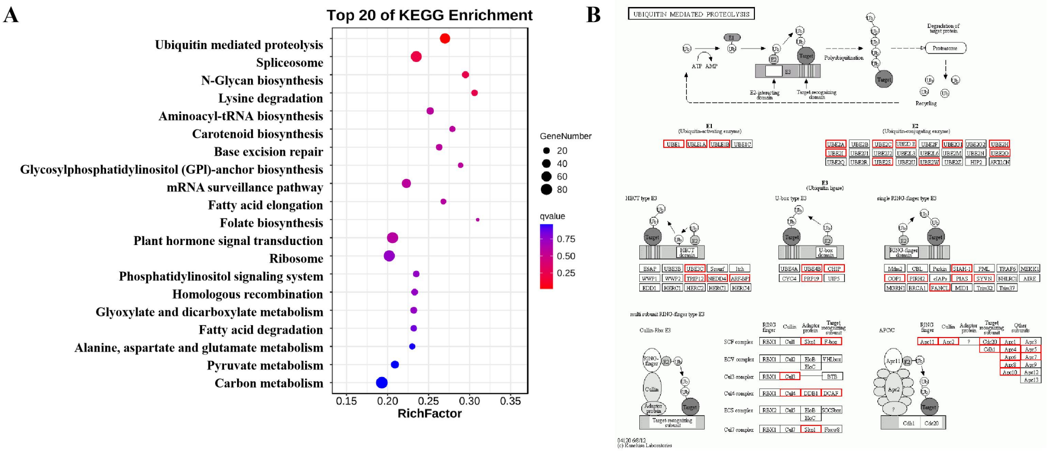

Figure S6 Enrichment analysis of all target genes regulated by DlERF6. **A** KEGG enrichment analysis of DlERF6 target genes. **B** The map of Ubiquitin mediated

proteolysis (ko04120) . The content within the red outlines indicated the target genes of DlERF6 identified in Ubiquitin-mediated proteolysis.

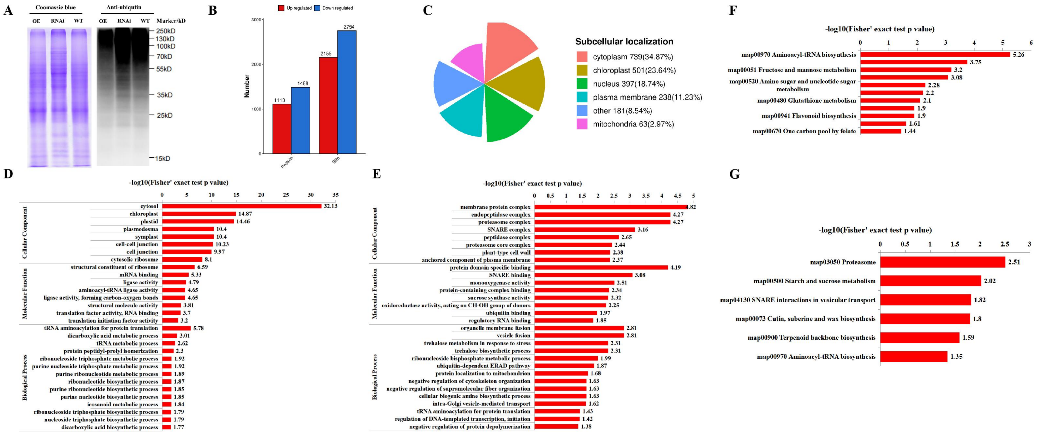

Figure S7 Overview of ubiquitylation in DlERF6-mediated resistance to heat stress

A Western blotting analysis of total ubiquitinated proteins in WT, *DlERF6* transgenic roots under heat stress. Coomassie blue stains are shown as loading controls. **B** The number of differentially expressed ubiquitylation sites and proteins in *DlERF6* overexpression (OE) vs WT roots under heat stress (n=3). **C** The subcellular localization analysis of differentially expressed proteins. **D-E** GO enrichment analysis of up- and down-regulated ubiquitinated proteins in *DlERF6* overexpression (OE) roots under heat stress, respectively. **F-G** KEGG enrichment analysis of up- and down-regulated ubiquitinated proteins in *DlERF6* overexpression (OE) roots under heat stress, respectively.

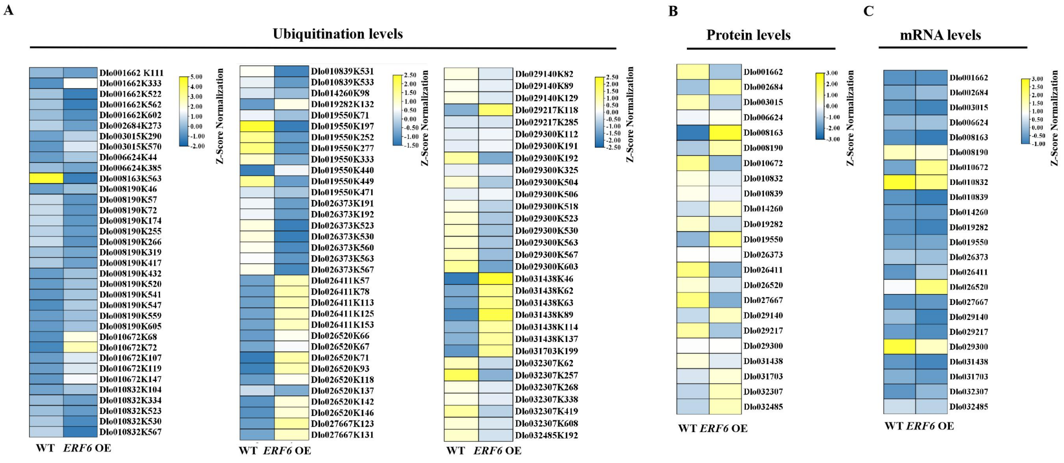

Figure S8 Comparative analysis of ubiquitination and expression patterns of HSP proteins in *DlERF6* Overexpression (OE) vs Wild-Type. **A** Heatmap comparison of the differentially Ubiquitinated proteins and sites of HSP proteins in *DlERF6*

overexpression (OE) vs Wild-Type (WT) roots. **B** Protein levels of the selected differentially ubiquitinated HSP proteins. **C** mRNA levels of the selected differentially ubiquitinated HSP proteins.

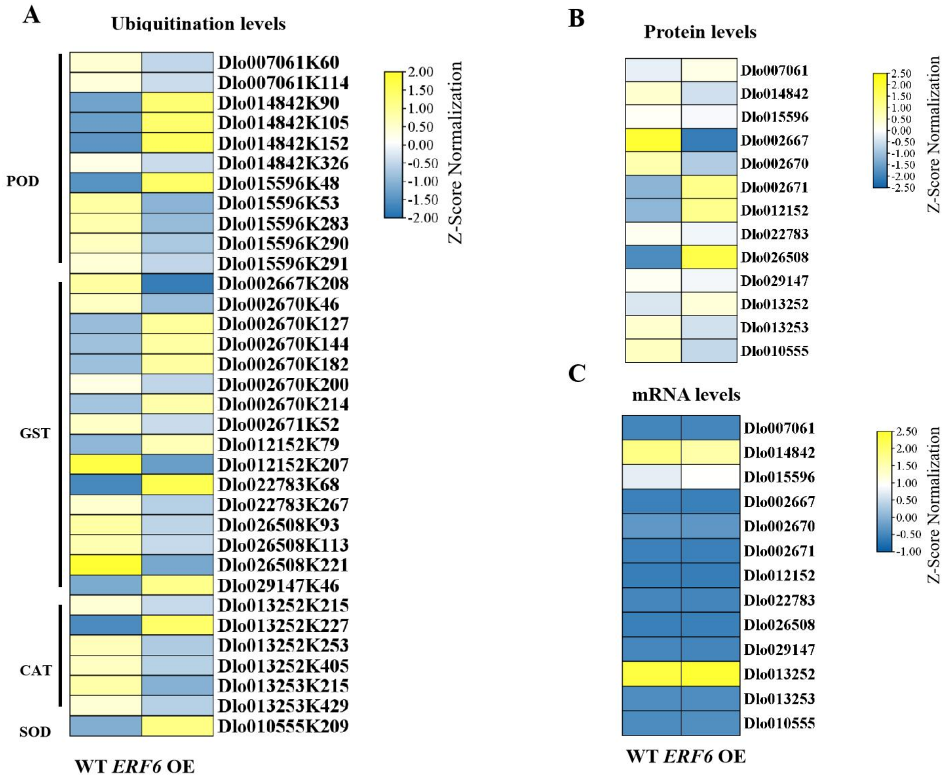

Figure S9 Comparative analysis of ubiquitination and expression patterns of ROS-related proteins in DlERF6 Overexpression (OE) vs Wild-Type (WT) Root. **A** Heatmap comparison of the differentially Ubiquitinated proteins and sites of ROS related proteins in *DlERF6* overexpression (OE) vs Wild-Type (WT) roots. **B** Protein levels of the selected differentially ubiquitinated ROS related proteins. **C** mRNA levels of the selected differentially ubiquitinated ROS related proteins.

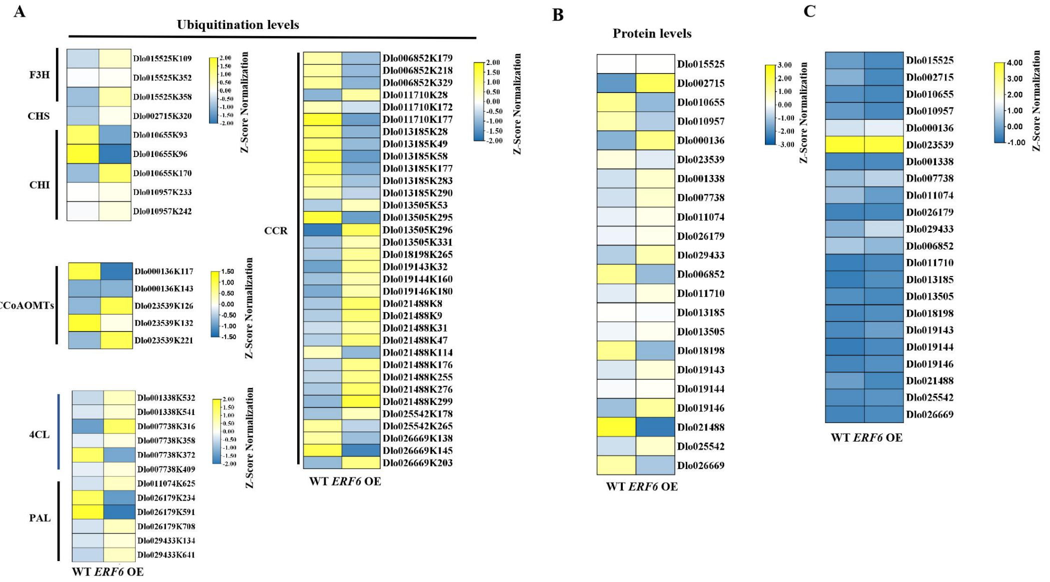

Figure S10 Comparative analysis of ubiquitination and expression patterns of flavonoid and lignin biosynthesis-related Proteins in *DlERF6* Overexpression (OE) vs Wild-Type (WT) Roots. A Heatmap comparison of the differentially Ubiquitinated proteins and sites of flavonoid and lignin biosynthesis related proteins in *DlERF6* overexpression (OE) vs Wild-Type (WT) roots. **B** Protein levels of the selected differentially ubiquitinated flavonoid and lignin biosynthesis related proteins. **C** mRNA levels of the selected differentially ubiquitinated flavonoid and lignin biosynthesis related proteins.

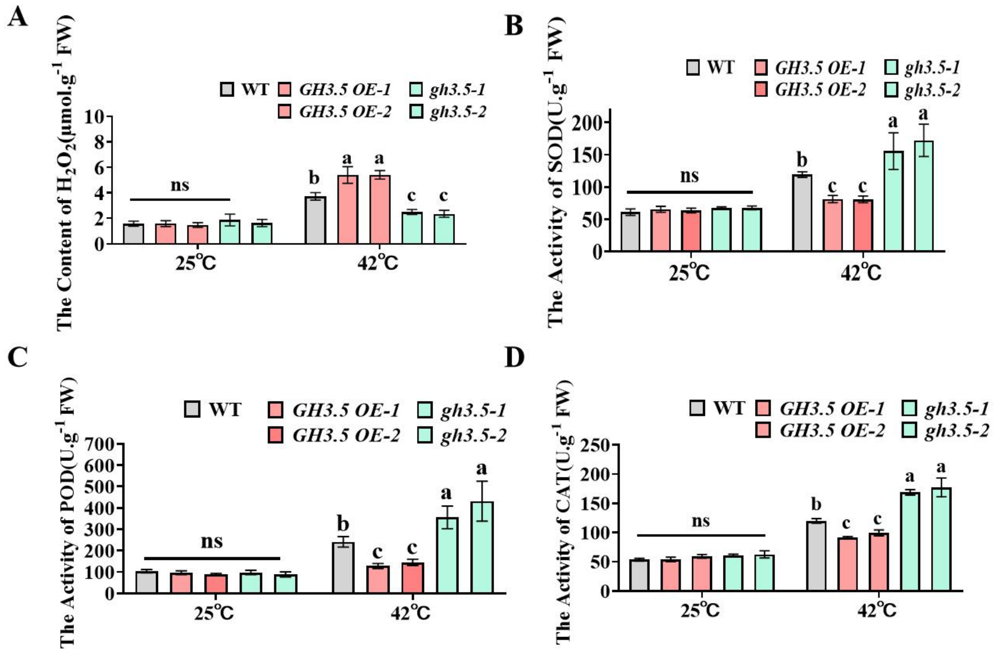

Figure S11 Effects of heat stress on H_2O_2 content and antioxidant enzyme activities in Wild-Type (WT) and *DlGH3.5* Transgenic Plants. A The H_2O_2 content of Wild-Type (WT), *DlGH3.5* transgenic plants leaves under normal and heat treatment. One way ANOVA was performed. Error bars represent the SD of mean values (n=3), and significant differences ($P < 0.05$) between groups are indicated by 'a','b' and 'c'. **B-D** The SOD, POD and CAT activities of Wild-Type (WT), *DlGH3.5* transgenic plants leaves under normal and heat treatment. One way ANOVA was performed. Error bars represent the SD of mean values (n=3), and significant differences ($P < 0.05$) between groups are indicated by 'a','b' and 'c'.
