# Supplementary figures and images for: The ETHYLENE RESPONSE FACTOR6-GRETCHEN HAGEN3.5 module regulates rooting and heat tolerance in Dimocarpus longan
Source: Plant Physiol. 2025 Mar 19;197(3):kiaf096. doi: 10.1093/plphys/kiaf096 (PMC11950727; doi:10.1093/plphys/kiaf096)

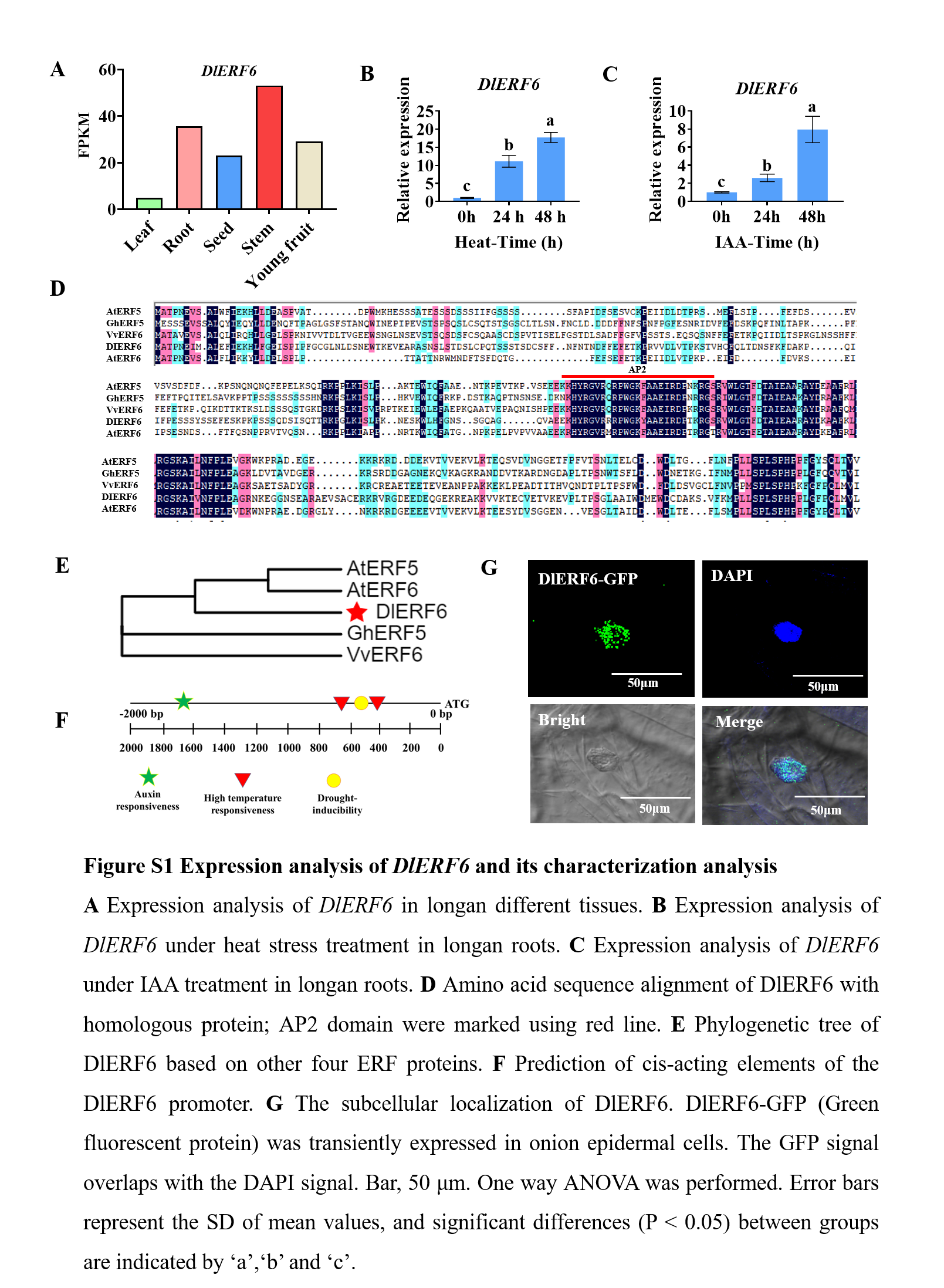

Supplement: kiaf096_Supplementary_Data [file kiaf096_supplementary_data.zip › FigureS1.png]

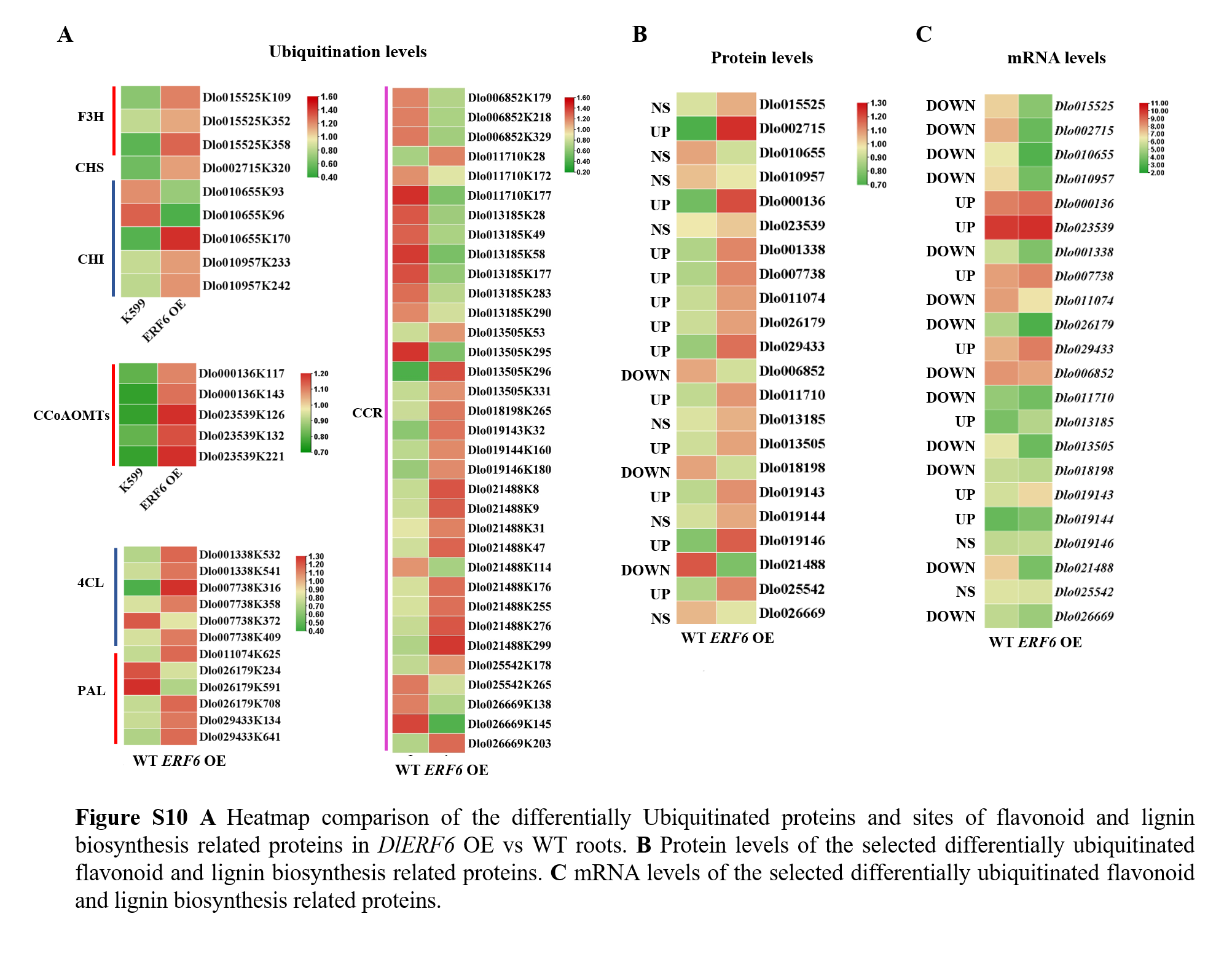

Supplement: kiaf096_Supplementary_Data [file kiaf096_supplementary_data.zip › FigureS10.png]

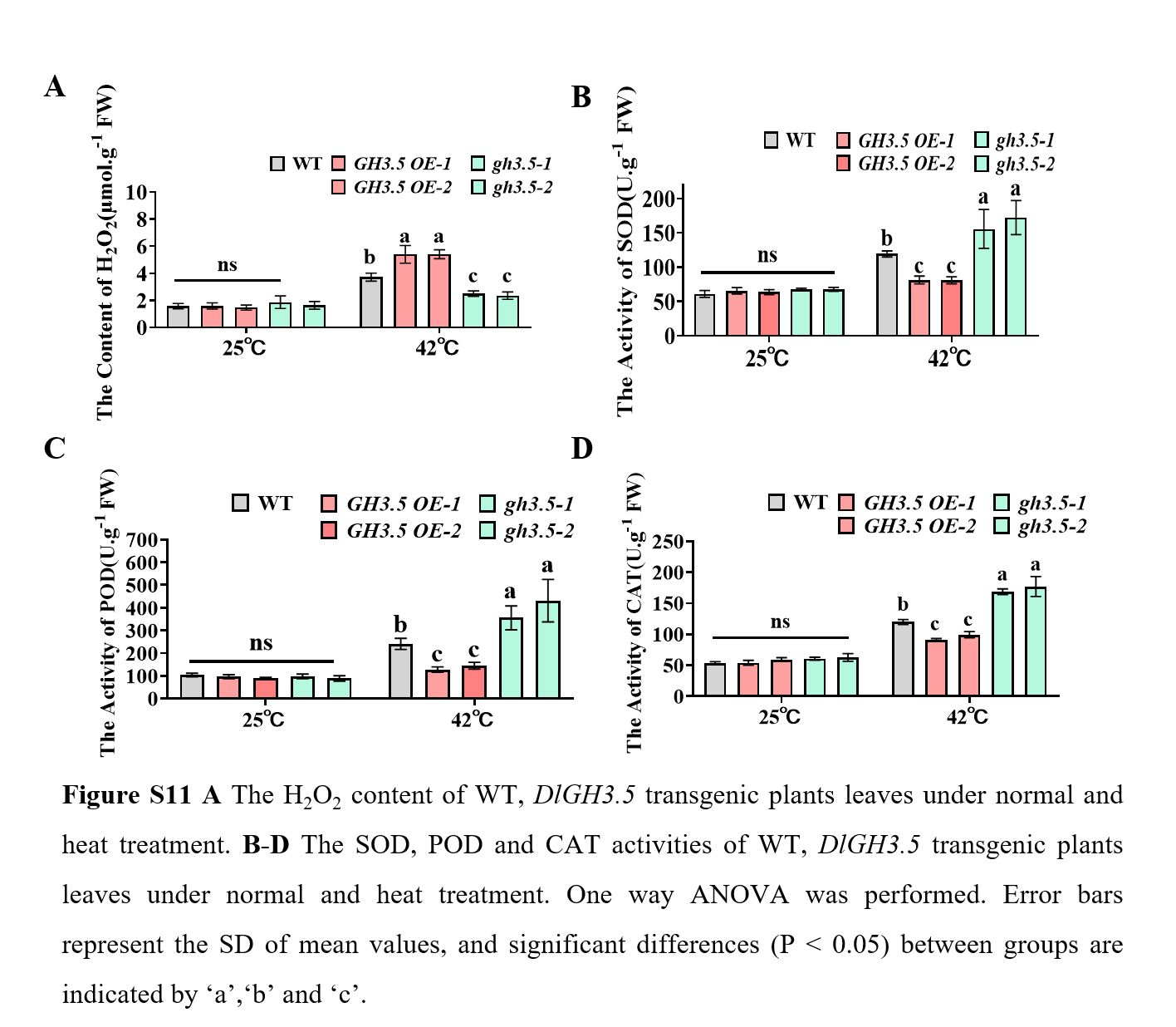

Supplement: kiaf096_Supplementary_Data [file kiaf096_supplementary_data.zip › FigureS11.png]

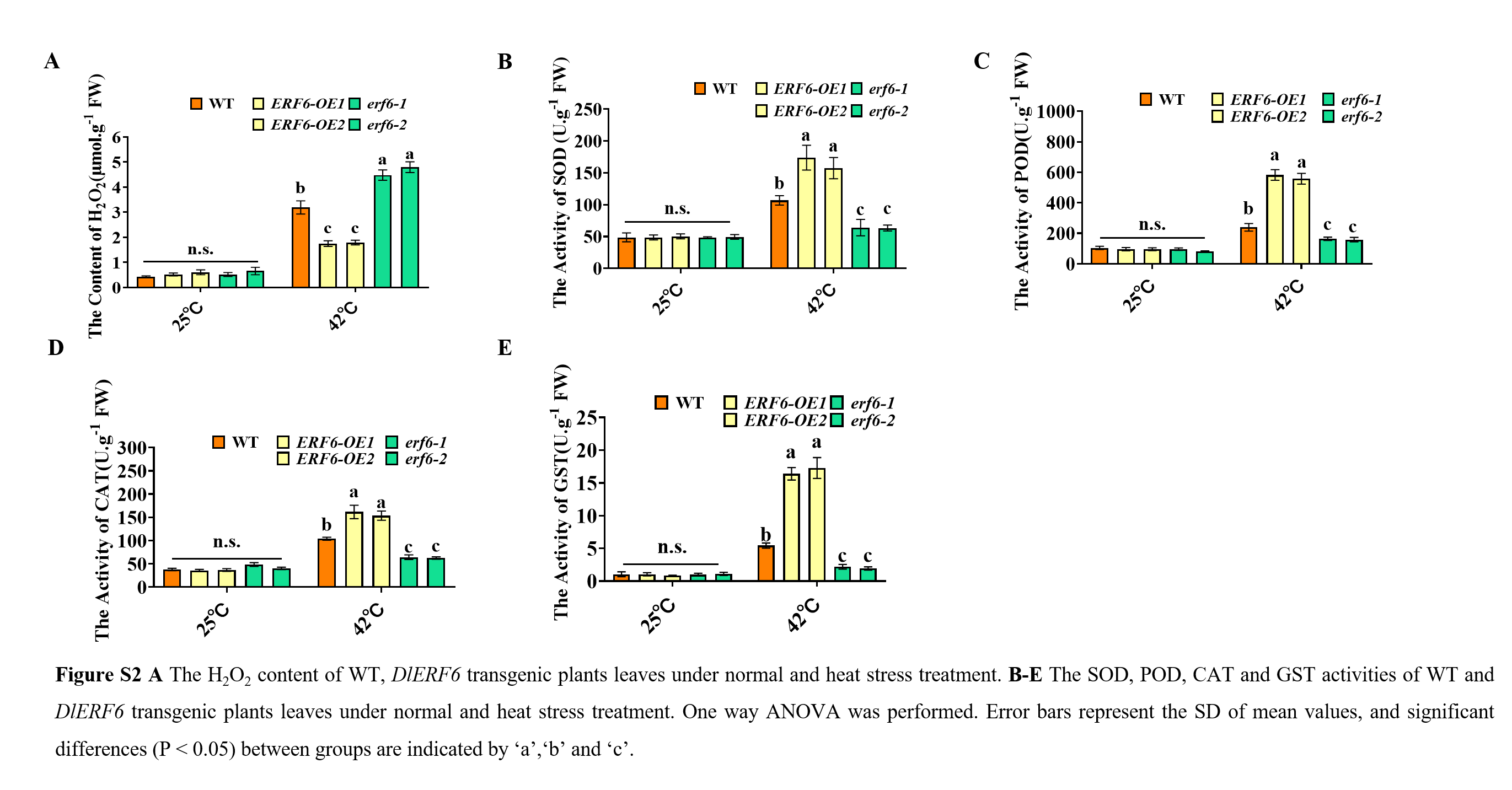

Supplement: kiaf096_Supplementary_Data [file kiaf096_supplementary_data.zip › FigureS2.png]

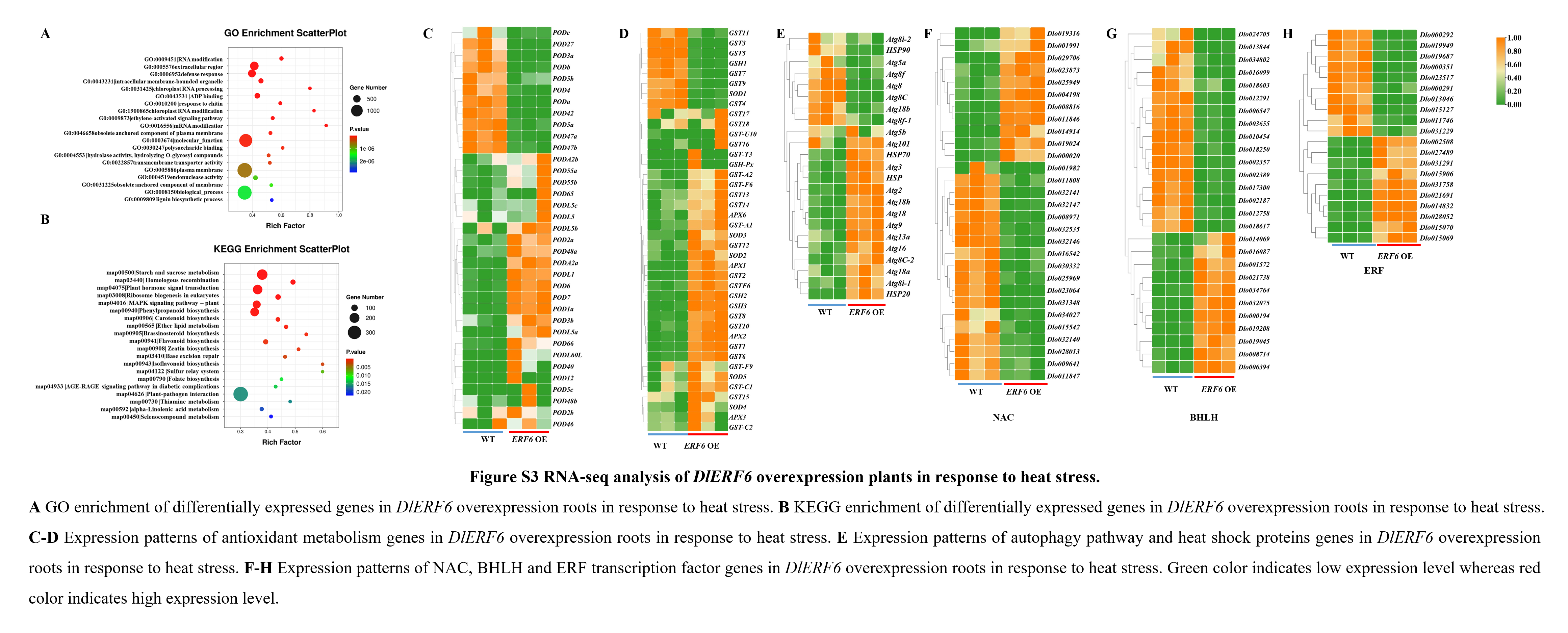

Supplement: kiaf096_Supplementary_Data [file kiaf096_supplementary_data.zip › FigureS3.png]

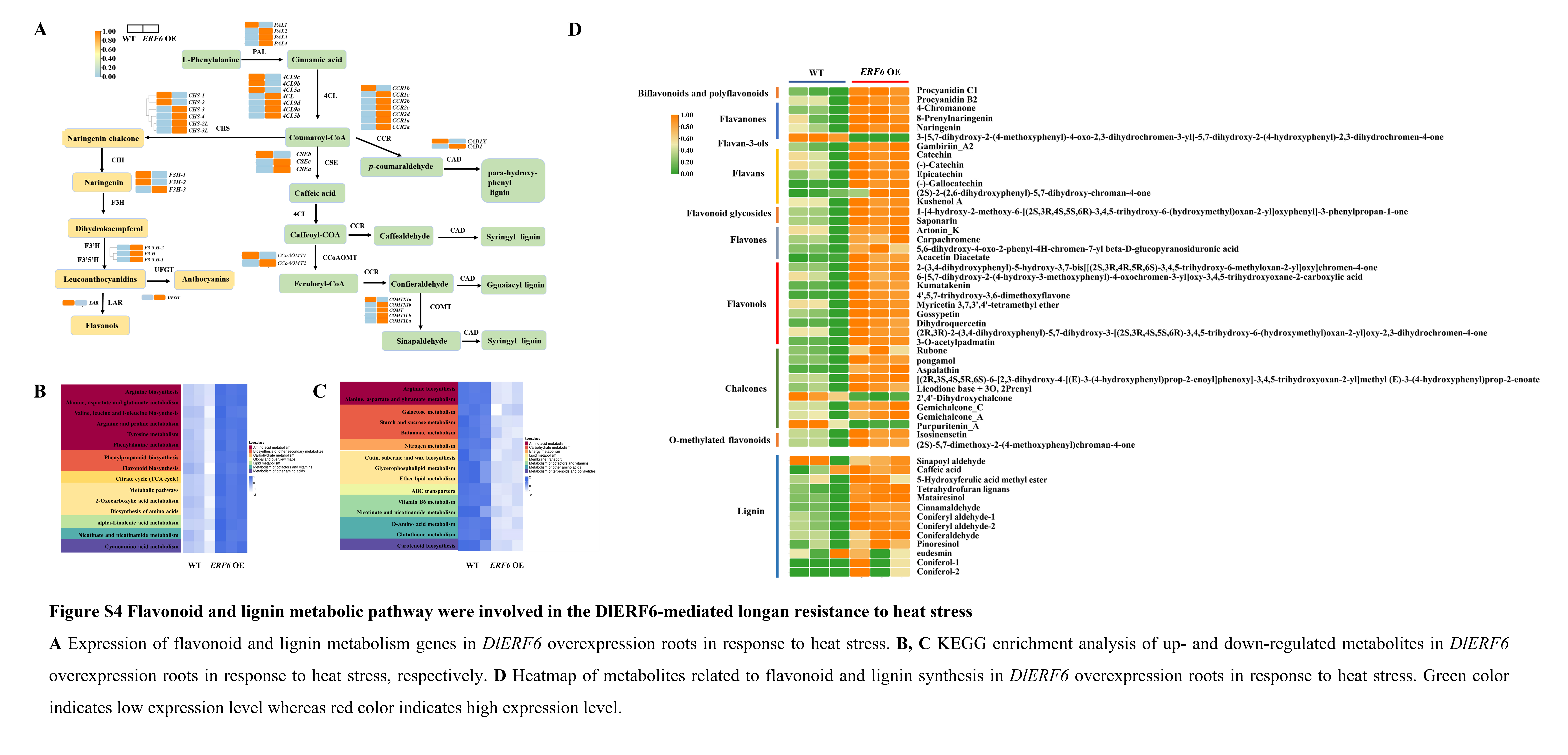

Supplement: kiaf096_Supplementary_Data [file kiaf096_supplementary_data.zip › FigureS4.png]

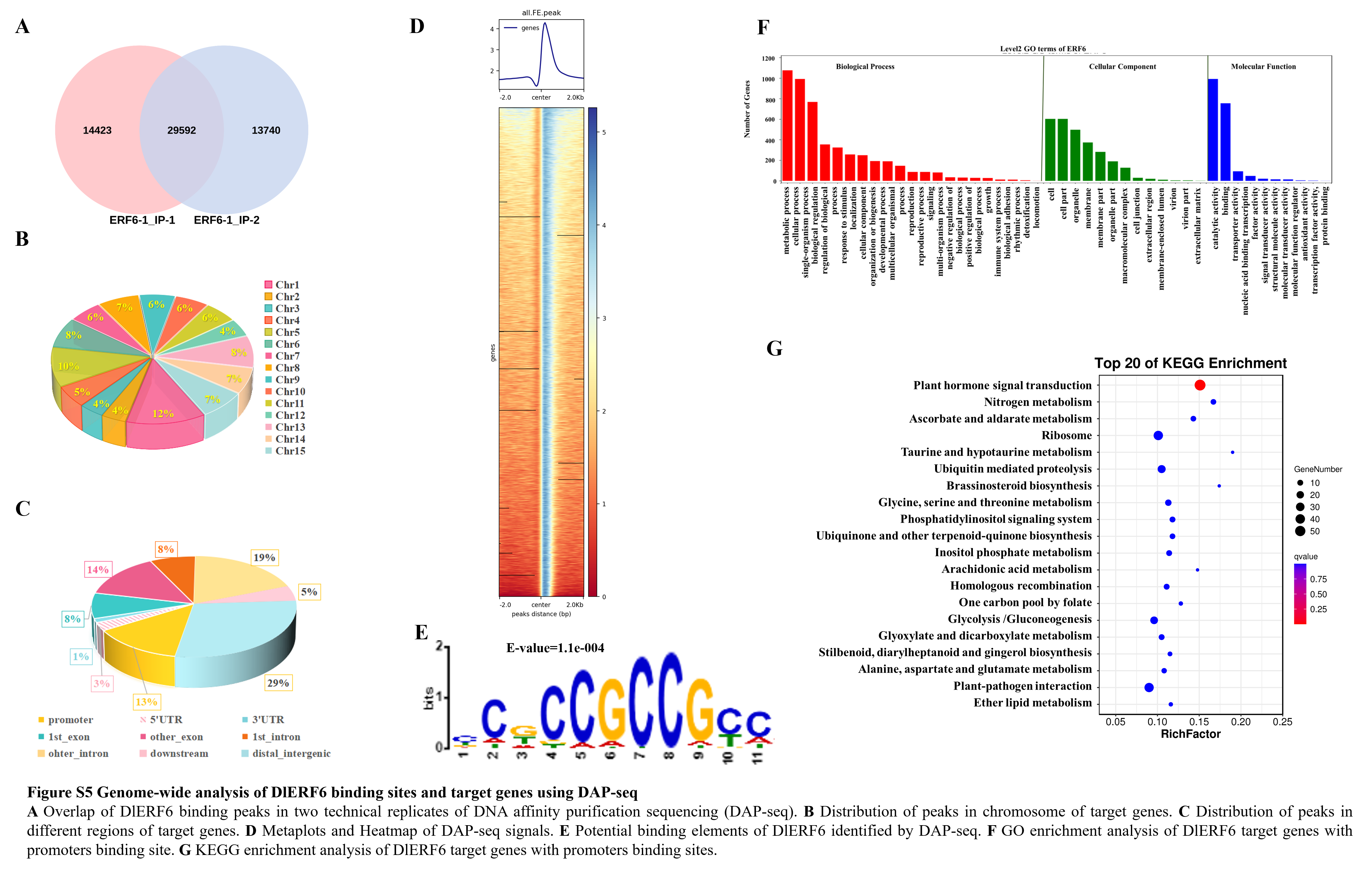

Supplement: kiaf096_Supplementary_Data [file kiaf096_supplementary_data.zip › FigureS5.png]

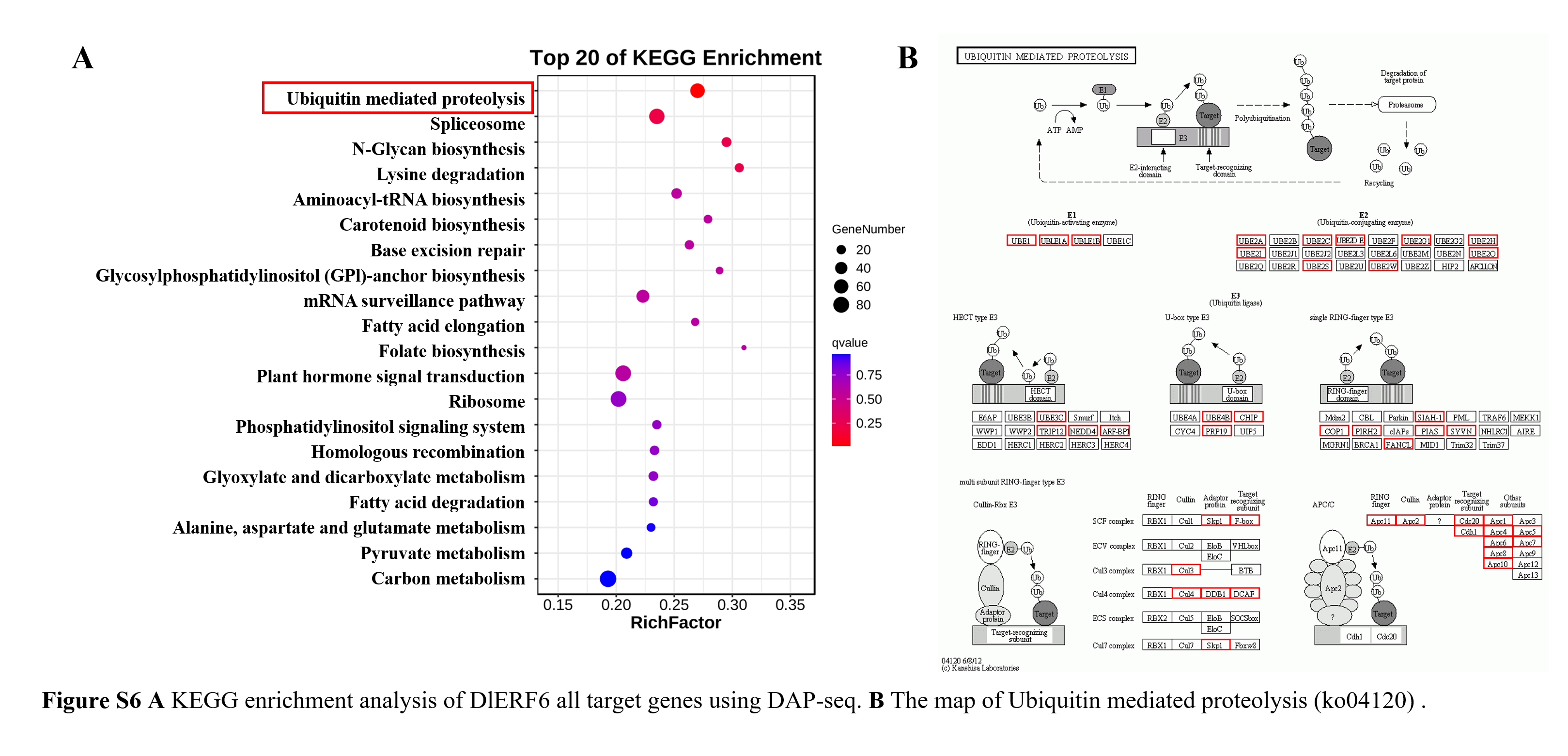

Supplement: kiaf096_Supplementary_Data [file kiaf096_supplementary_data.zip › FigureS6.png]

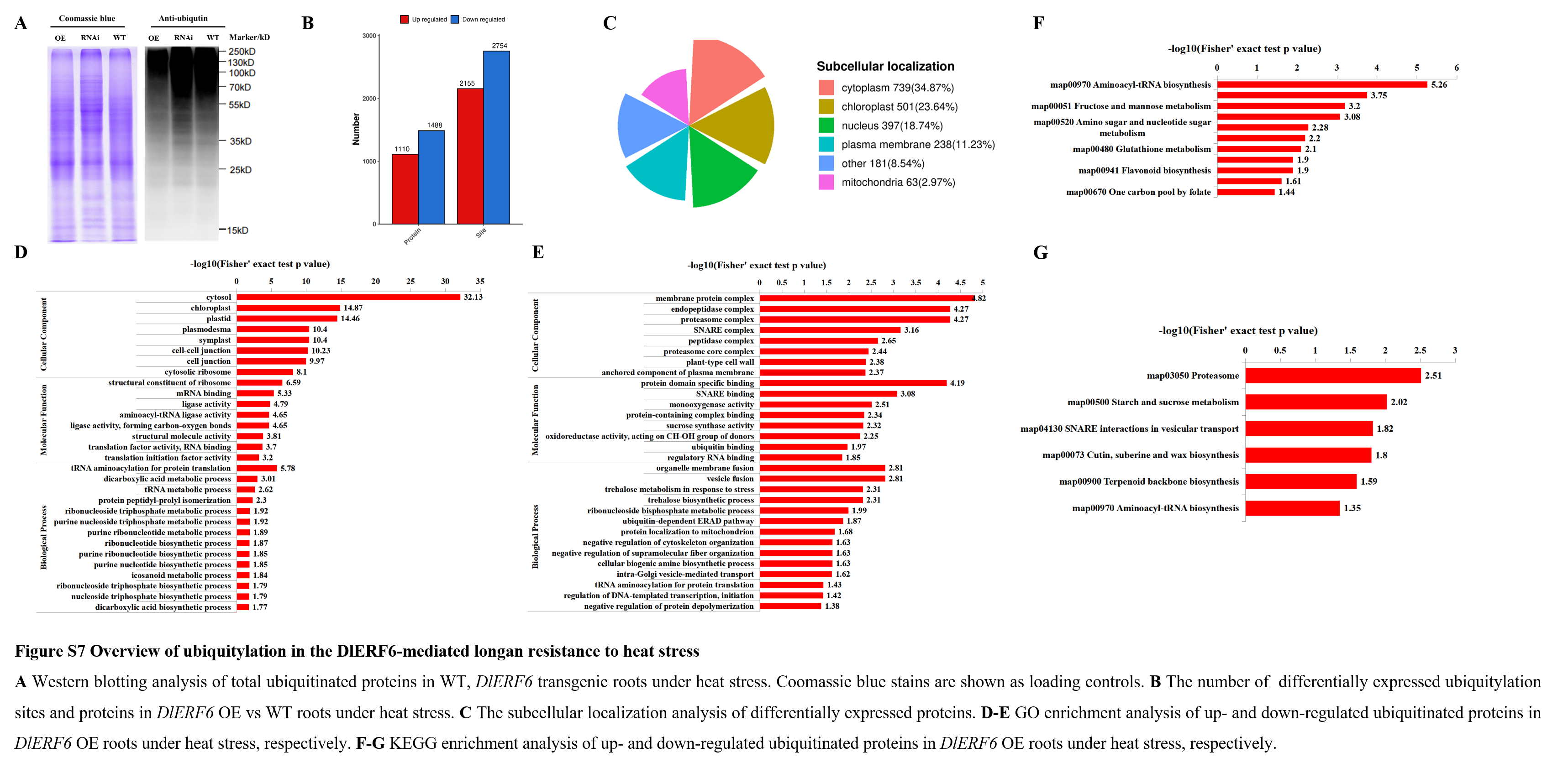

Supplement: kiaf096_Supplementary_Data [file kiaf096_supplementary_data.zip › FigureS7.png]

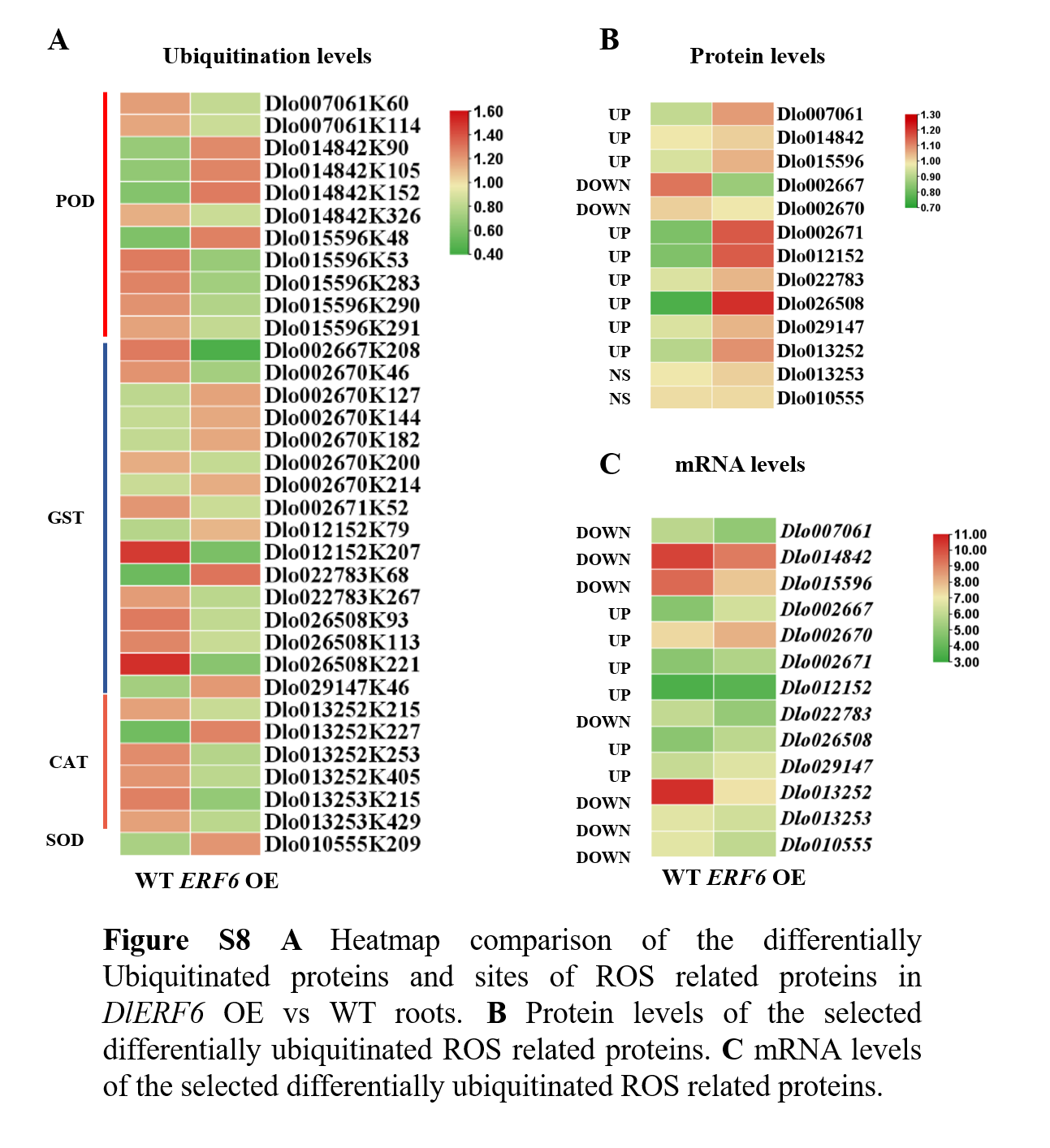

Supplement: kiaf096_Supplementary_Data [file kiaf096_supplementary_data.zip › FigureS8.png]

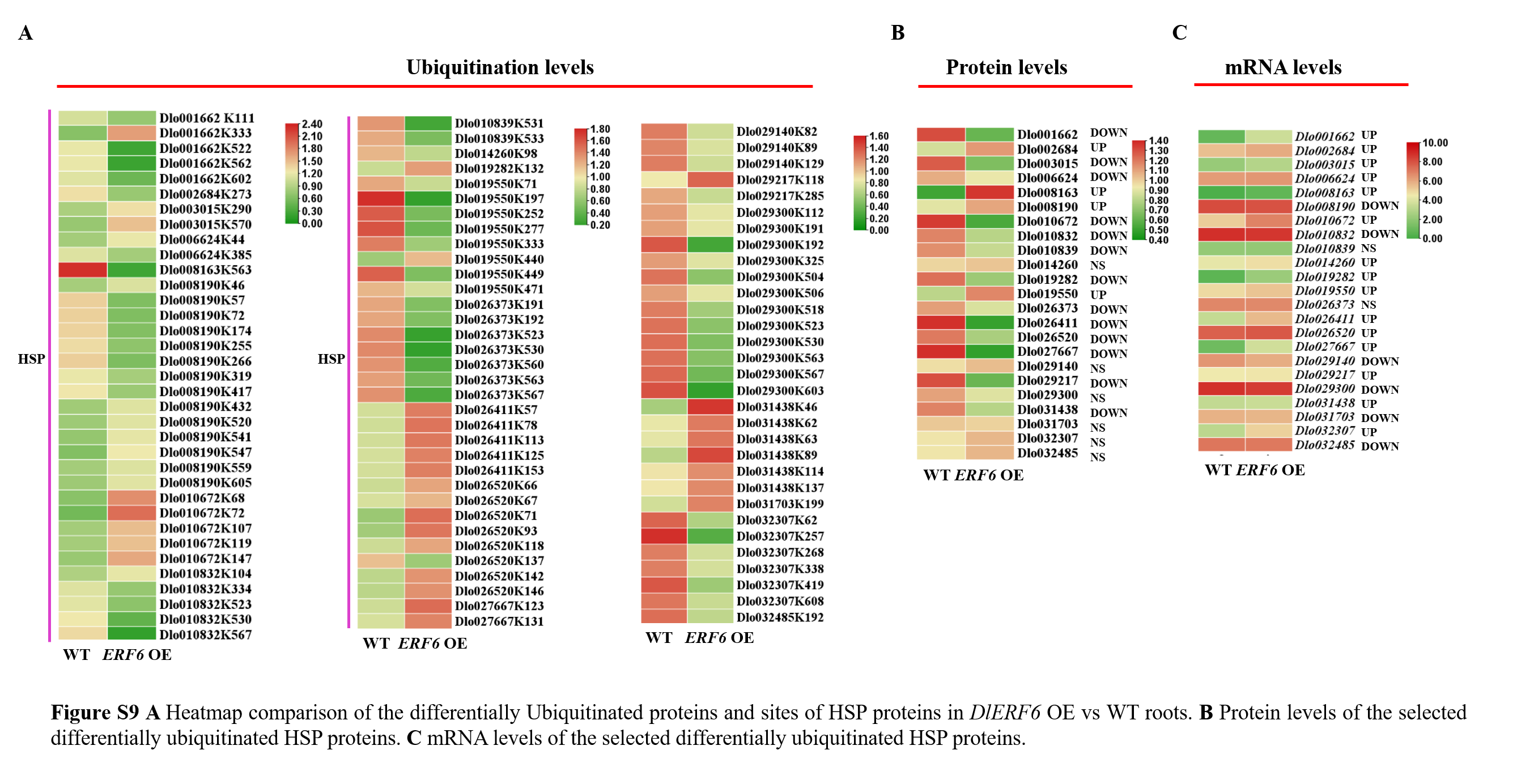

Supplement: kiaf096_Supplementary_Data [file kiaf096_supplementary_data.zip › FigureS9.png]
